# Supplementary material for: Zika virus induces FOXG1 nuclear displacement and downregulation in human neural progenitors
Source: Stem Cell Reports. 2022 Jun 16;17(7):1683–98. doi: 10.1016/j.stemcr.2022.05.008 (PMC9287670; doi:10.1016/j.stemcr.2022.05.008)
Supplement: Document S1. Figures S1–S6 and Experimental procedures [file mmc1.pdf]

Stem Cell Reports, Volume *17*

Supplemental Information

Zika virus induces FOXG1 nuclear displacement and downregulation in human neural progenitors

Giulia Lottini, Matteo Baggiani, Giulia Chesi, Beatrice D'Orsi, Paola Quaranta, Michele Lai, Laura Pancrazi, Marco Onorati, Mauro Pistello, Giulia Freer, and Mario Costa

Supplemental items

Figure S1

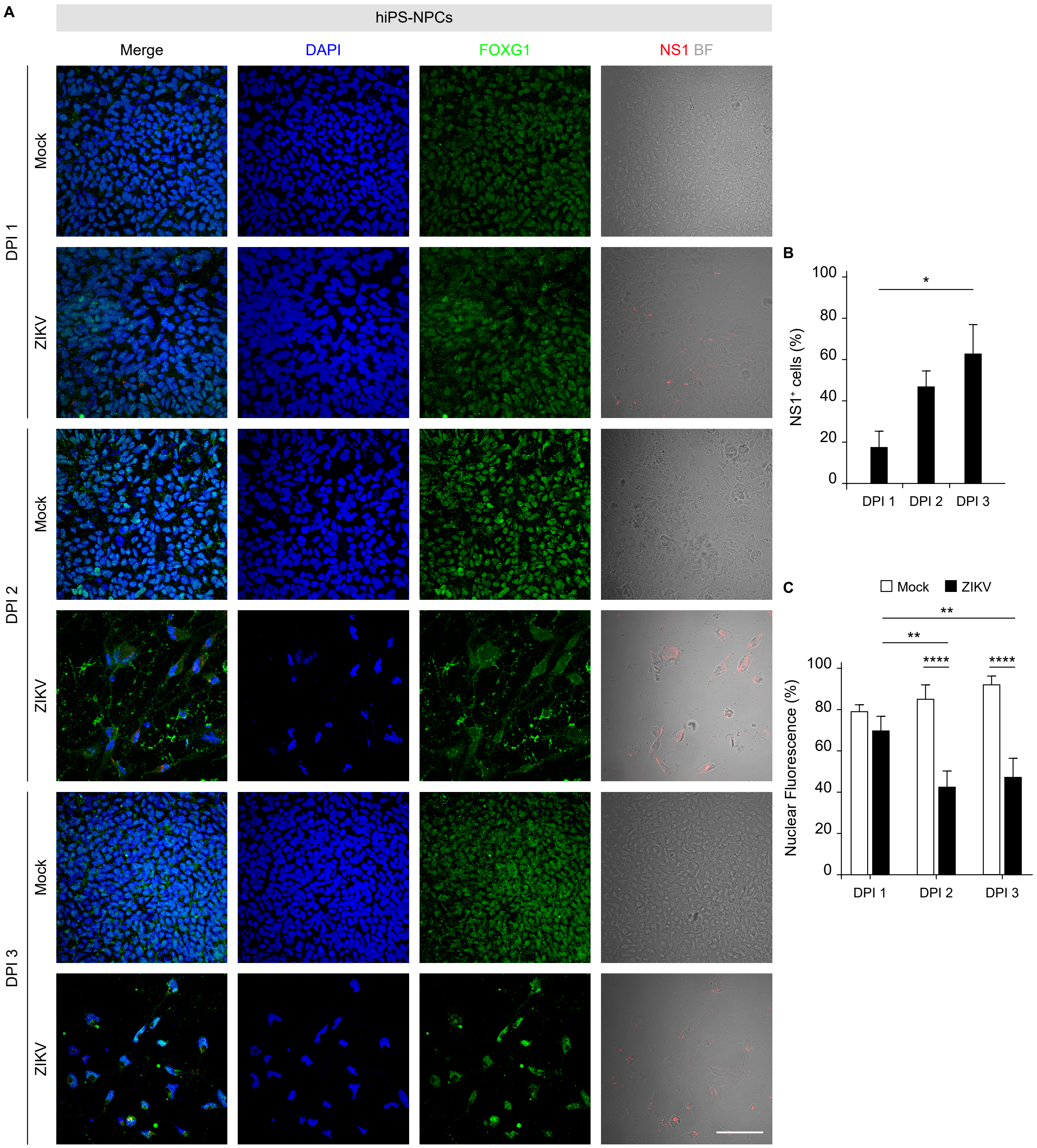

Figure S1. Infectivity rate and FOXG1 mislocalization in hiPS-NPCs after ZIKV infection, Related to Figure 1. (A) Representative confocal images of FOXG1, ZIKV NS1, Bright Field (BF), and DAPI in mock and ZIKV-infected hiPS-NPCs at day post-infection (DPI) 1, 2, and 3. The time-course analysis shows progressive ZIKV infection and FOXG1 mislocalization from DPI 1 to 3. Scale bar = 50 µm. (B) Bar plot indicating the rate of ZIKV infection at each DPI. Data are shown as mean ± SEM (total cells, n > 800), *p*-value < 0.05 (One-way ANOVA, *post hoc* Tukey's test). (C) Bar plot indicating the ratio of FOXG1 nuclear fluorescence on total fluorescence in mock and ZIKV-infected conditions at each DPI. Data are shown as mean ± SD (total cells, n = 120), *p*-value < 0.01 (Two-way ANOVA, *post hoc* Tukey's test).

Figure S2

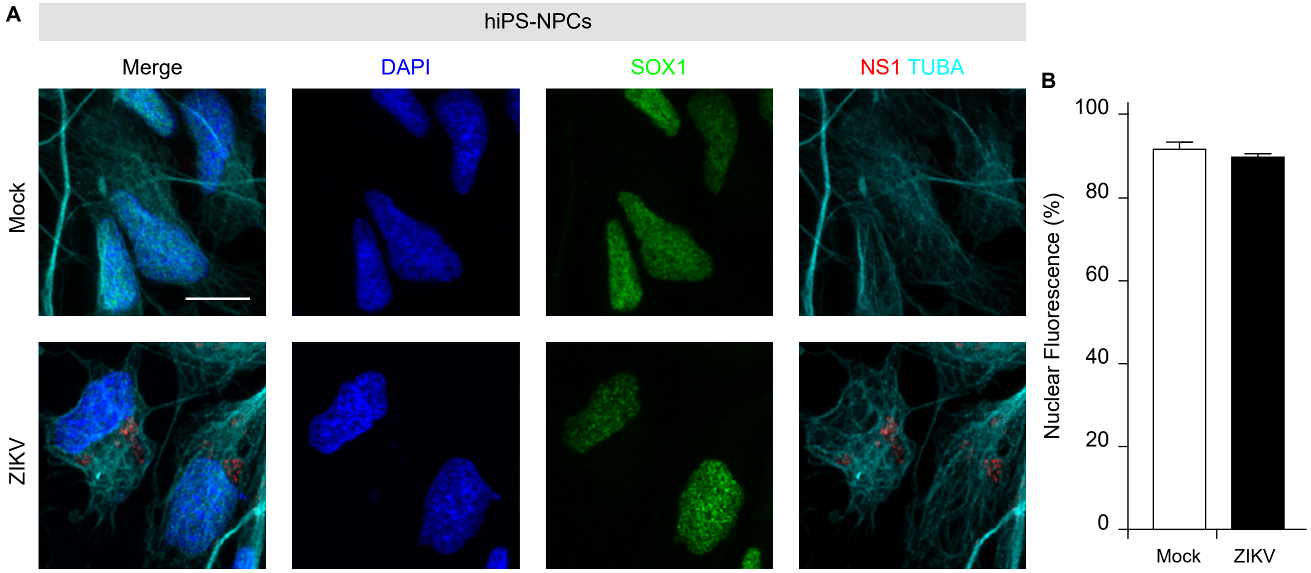

Figure S2. ZIKV infection does not induce mislocalization of SOX1 in hiPS-NPCs, Related to Figure 1. (A) Representative confocal images of SOX1, ZIKV NS1, TUBA (α-tubulin), and DAPI in mock and ZIKV-infected hiPS-NPCs. Scale bar = 10 µm. (B) Bar plot indicating the ratio of SOX1 nuclear fluorescence on total fluorescence in mock and ZIKV-infected conditions. Data are shown as mean ± SD (total cells, n = 40), *p*-value > 0.05 (unpaired Student's *t*-test).

Figure S3

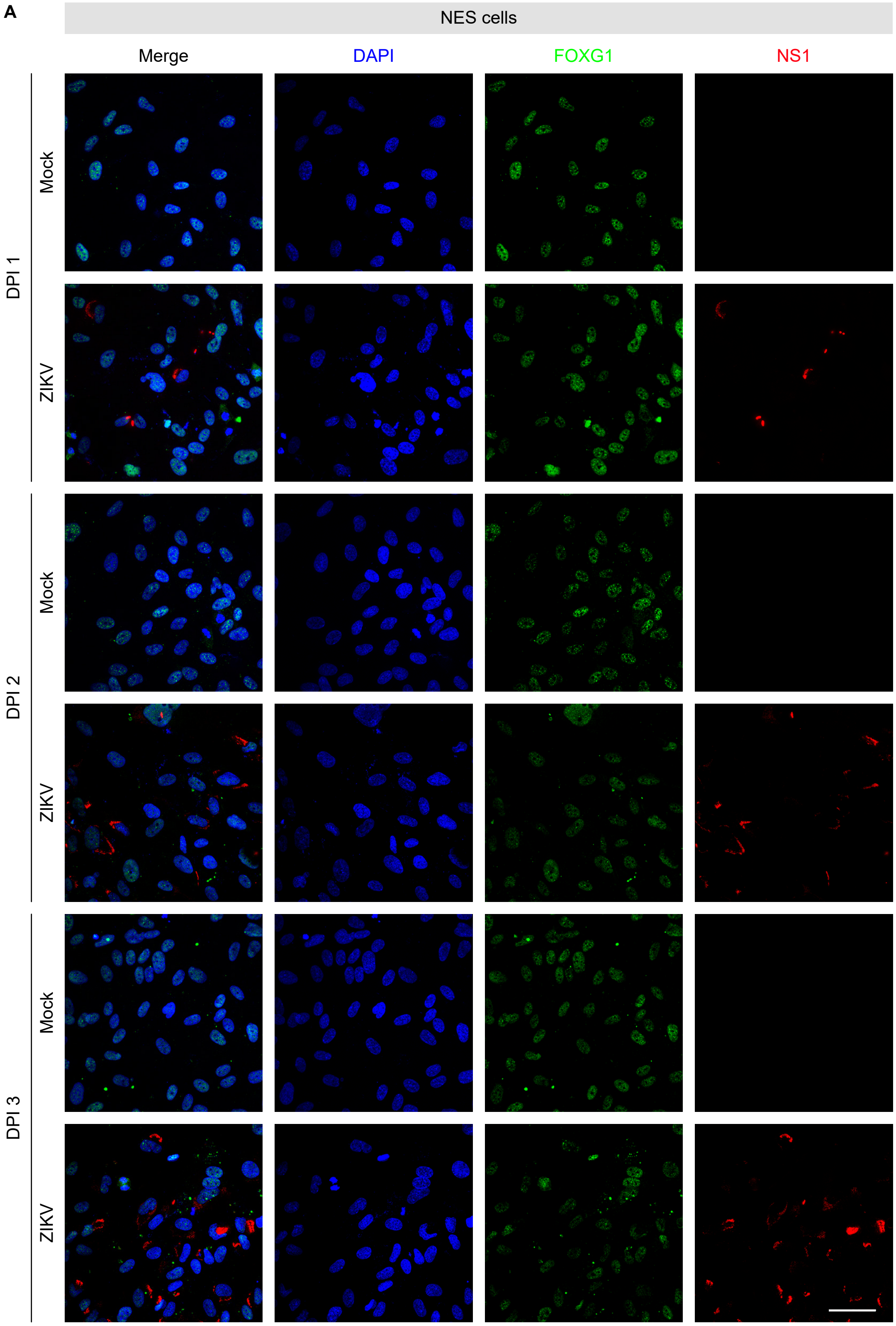

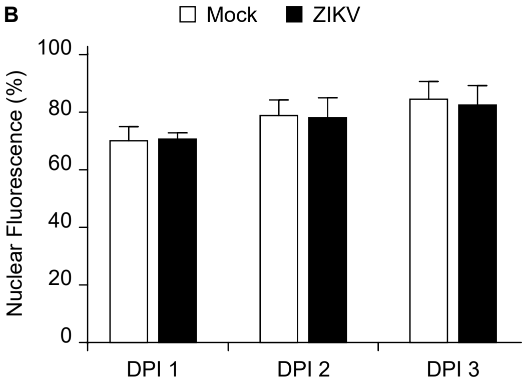

Figure S3. Time-course of FOXG1 localization pattern in NES cells after ZIKV infection, Related to Figure 3.
(A) Representative confocal images of FOXG1, ZIKV NS1, and DAPI in mock and ZIKV-infected NES cells at DPI 1, 2, and 3. The time course analysis shows that FOXG1 does not mislocalize from the nucleus. Scale bar = 50 µm. (B) Bar plot indicates the ratio of FOXG1 nuclear fluorescence on total fluorescence in mock and ZIKV-infected conditions at each DPI. Data are shown as mean ± SD (total cells, n = 120), *p*-value > 0.05 (Two-way ANOVA, *post hoc* Tukey's test).

Figure S4

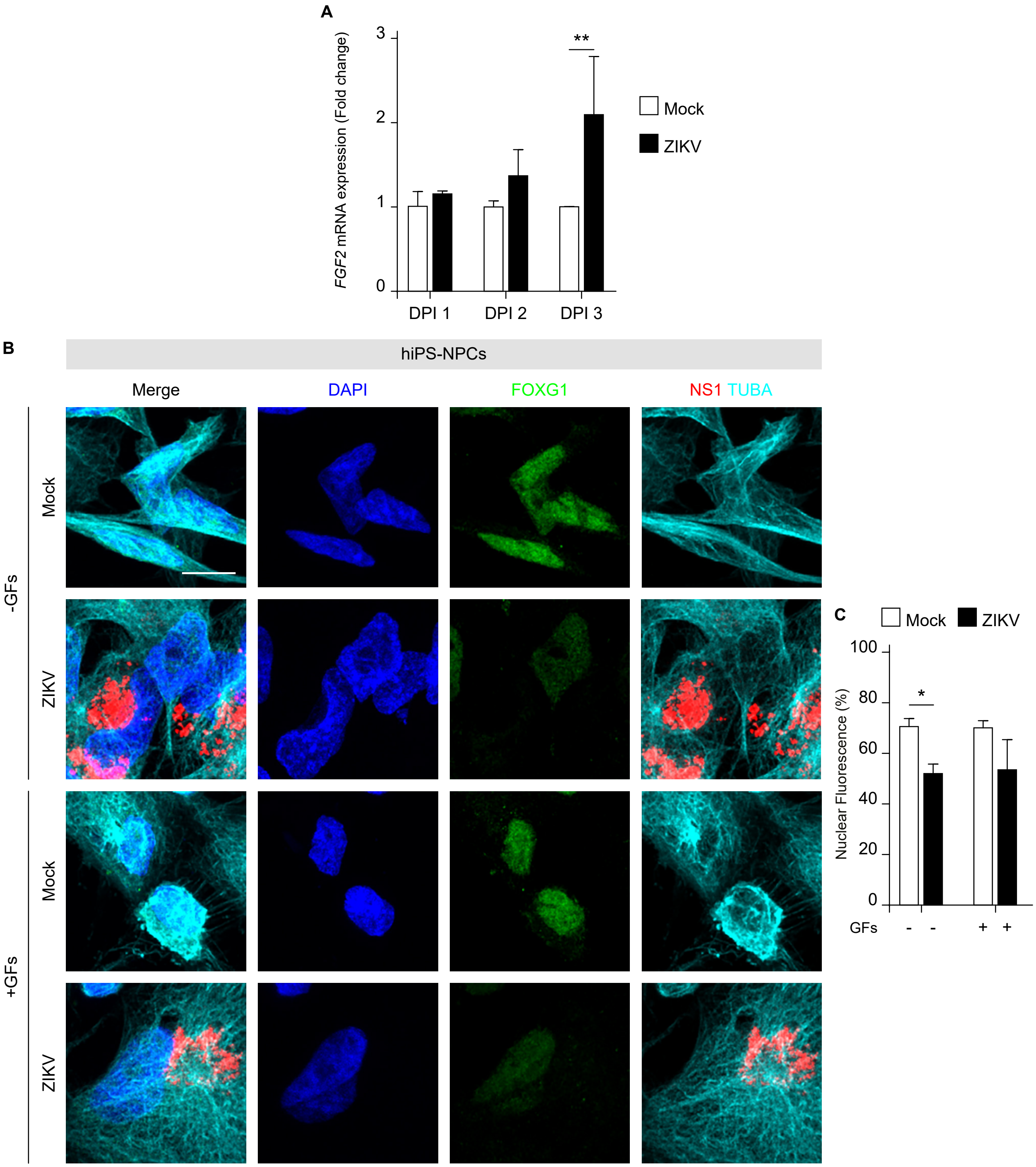

Figure S4. *FGF2* expression analysis and GF treatment after ZIKV infection in hiPS-NPCs, Related to Figure 3.
(A) Bar plot indicating fold change in *FGF2* mRNA level in mock and ZIKV-infected conditions. Data are shown as mean ± SD (n = 3), *p*-value < 0.01 (Two-way ANOVA, *post hoc* Tukey's test). (B) Representative confocal images of FOXG1, ZIKV NS1, TUBA (α-tubulin), and DAPI in mock and ZIKV-infected hiPS-NPCs in the absence and in the presence of GFs (EGF and FGF2) after the infection, in which FOXG1 preserves nuclear localization following ZIKV infection only in presence of GFs. Analyses were performed at DPI 3. Scale bar = 10 µm. (C) Bar plot indicating the ratio of FOXG1 nuclear fluorescence on total fluorescence in mock and ZIKV-infected conditions at DPI 3. Data are shown as mean ± SD (total cells, n = 80), *p*-value < 0.05 (Two-way ANOVA, *post hoc* Tukey's test).

Figure S5

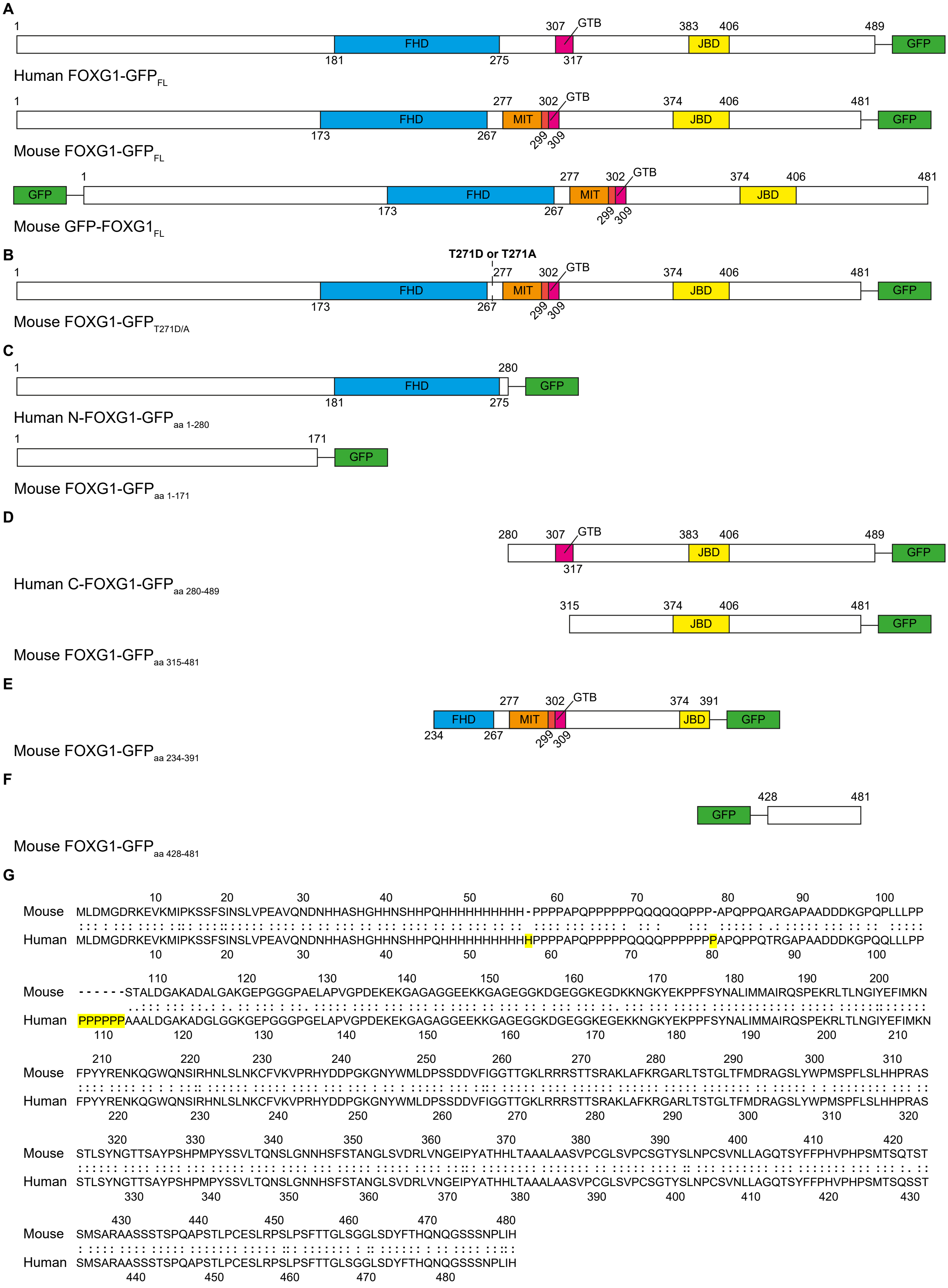

Figure S5. Human and mouse FOXG1-GFP constructs, Related to Figures 4 and 5.
Schematic illustration of (A) human FOXG1-GFP full length (FL), mouse FOXG1-GFP FL and mouse GFP-FOXG1 FL; (B) mutated mouse FOXG1-GFP on Thr271 (T271D/A); (C) N-terminal human FOXG1-GFP (1-280) and N-terminal mouse FOXG1-GFP (1-171); (D) C-terminal human FOXG1-GFP (280-489) and C-terminal mouse FOXG1-GFP (315-481); (E) central domains of mouse FOXG1-GFP (234-391) (F) final part of C-terminal mouse FOXG1-GFP (428-481). (G) Comparison between mouse and human aa sequences of FOXG1; the human-specific aa in FOXG1 protein are highlighted in yellow. FHD = Forkhead Domain (blue); MIT = Mitochondrial domain (orange); GTB = GROUCHO/TLE-Binding domain (pink); JBD = JARID1B Binding Domain (yellow); GFP = Green Fluorescence Protein (green).

Figure S6

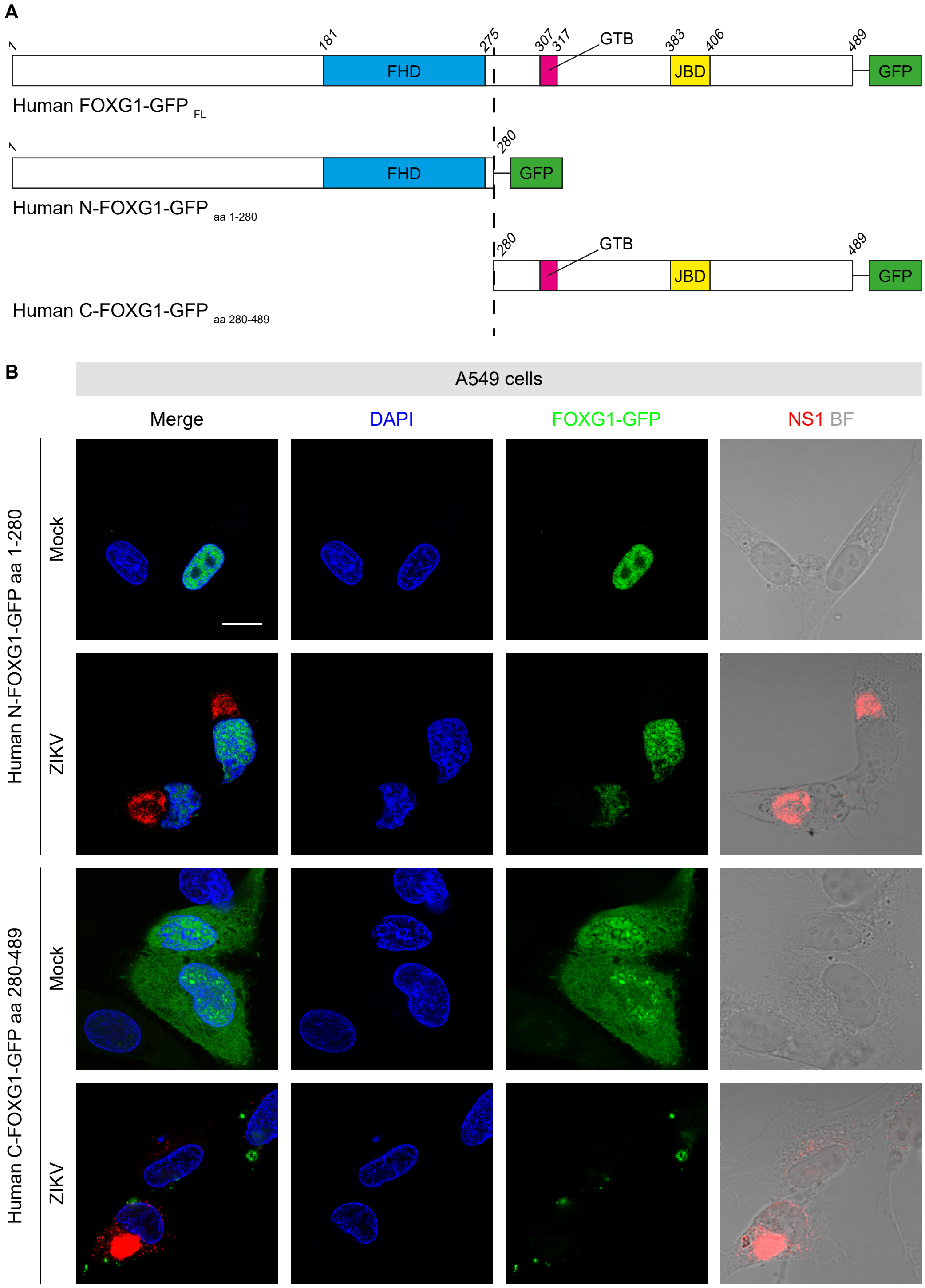

Figure S6. Human FOXG1 C-terminus is essential for reacting to ZIKV infection, Related to Figure 5.
(A) Schematic illustration of FOXG1-GFP full length (FL) and the partial constructs (human N-FOXG1-GFP aa 1-280; human C-FOXG1-GFP aa 280-489). FHD = Forkhead Domain (blue); GTB = GROUCHO/TLE-Binding domain (pink); JBD = JARID1B Binding Domain (yellow); GFP = Green Fluorescence Protein (green). (B) Representative confocal images of human N-FOXG1-GFP aa 1-280 and human C-FOXG1-GFP aa 280-489 transfected A549 cells in mock and ZIKV-infected conditions. BF = Bright field. Analyses were performed at DPI 1. Scale bar = 20 μm.

Supplemental experimental procedures

hiPS-NPC maintenance and derivation

We derived neural progenitor cells from hiPSCs, as previously reported (1). Briefly, hiPSCs were dissociated into single cells in StemFlex medium (Thermo Fisher Scientific; #A3349201)] in Matrigel coated dishes containing 10 μM Y-27632, until confluent. Then, we performed the dual SMAD inhibition protocol changing the StemFlex medium with a neural induction medium [1:1 Dulbecco's minimum essential medium/F12 (DMEM/F12) (Gibco #11330-032) and Neurobasal medium (Gibco #21103-049) with addition of B27 supplement (1:50, Gibco, #175040-44), N2 supplement (1:100, Gibco, #17502-048), 20 μg/ml insulin (Sigma, # I9278), L-glutamine (1:100, Gibco, #25030-081), MEM Non-Essential Amino Acids (1:100, Gibco, #11140-050) and 2-mercaptoethanol (1:1000, Gibco, #21985)], supplemented with 100 nM of LDN-193189 (StemCell Technologies, # 72144), 10 μM of SB-431542 (Merck, # 616464-5MG) and 2 μM of XAV939 (StemCell Technologies, # 72674). The medium was changed daily until day 11. At day 12, the cells were dissociated with Accutase and maintained in a neural differentiation medium [Neurobasal medium (Gibco #21103-049) with addition of B27 supplement (1:50, Gibco, #175040-44), N2 supplement (1:100, Gibco, #17502-048), L-glutamine (1:100, Gibco, #25030-081), with Y-27632 (10 μM), to increase cell viability.

Maintenance of NES cells

Human neuroepithelial stem (NES) cells were cultured as already reported (2,3). Briefly, NES cells were maintained in NES medium [Dulbecco's minimum essential medium/F12 (DMEM/F12) (Gibco #11330-032) with addition of B27 supplement (1:1000, Gibco, #175040-44), N2 supplement (1:100, Gibco, #17502-048), 20 ng/ml FGF-2 (Gibco, #13256029), 20 ng/ml EGF (Gibco, #PHG0311), 1.6 g/l glucose, 20 μg/ml insulin (Sigma, # I9278) and 5 ng/ml BDNF (Gibco, #PHC7074)] in poly-L-ornithine (0.01%, Sigma, #P4957), laminin (5 μg/ml, Invitrogen #23017-015) and fibronectin (1μg/ml, Corning, #354008) coated dishes. In order to preserve their optimal growth and neurogenic properties, the medium should be changed every 2-3 days and the cells should be passaged 1:2-3 when they are confluent, once every 5-7 days (~0.5–1 x 105 cells/cm2).

Maintenance of A549 cells

A549 were grown in DMEM, high glucose, 1 mM glutamine, 10% FCS, unless otherwise stated. No antibiotics were added. All the cells were routinely tested for mycoplasma (4).

DNA constructs

Mouse *Foxg1* aa 1-171cDNA was amplified by PCR using *GFP-Foxg1* wt as DNA template and a forward primer incorporating the *XhoI* restriction site that occurs in the *Foxg1* cDNA (underlined) Forward primer: 5'-ACTCGAGCATGCTGGACATGGGAGATAGG-3'.
The reverse primer was 5'GGATCCCCATGTATTAAAGGGTTGGAAG-3', incorporating a *BamHI* restriction site (underlined). The amplification product was purified and cleaved with *XhoI/BamHI* and ligated to the corresponding restriction sites in the vector pEGFP-N1 (Clontech, USA). FOXG1 aa 1-280-GFP, FOXG1 aa 280-489-GFP, FOXG1- T271D-GFP (phospho-mimetic T271-ACG were changed in Asp-GAC), FOXG1-T271A-GFP (phospho- defective T271-ACG were changed in Ala-GCG) were purchased at IDT, Belgium. Mouse *Foxg1* aa 315-489 cDNA was amplified by PCR using *GFP-Foxg1* as DNA template. The forward primer was 5'-GGTACCAATGAGCACTTTGAGTTACAACGG-3', incorporating a *KpnI* restriction site (underlined) and a start codon (bold) before the codon coding for amino acid 315. The reverse primer was 5'-GGATCCCCATGTATTAAAGGGTTGGAAG-3', incorporating a *BamHI* restriction site (underlined). The amplification product was purified and cleaved with *KpnI/BamHI* and ligated to the corresponding restriction sites in the vector pEGFP-N1 (Clontech, USA). The GFP-FOXG1 aa 428-481 construct was generated digesting *Foxg1-GFP* with *SmaI* and *BamHI.* The excised fragment was cloned in frame into the corresponding restriction sites of pEGFP-C2.

Immunofluorescence

Cultured cells were fixed in 4% formaldehyde (FA) for 12 min at RT. After two 3-minutes washed with Phosphate Buffered Saline-Triton X-100 (PBSX) [0.1% (vol/vol) Triton X-100 in PBS Ca2+/Mg2+ 1X] cells were permeabilized for 10 min with permeabilization solution [0.5% (vol/vol) Triton X-100 in PBS Ca2+/Mg2+1X] and blocked for 1 h with blocking solution [5% fetal bovine serum, 0.3% (vol/vol) Triton X-100 in PBS Ca2+/Mg2+1X]. Cells were incubated with primary antibodies against flavivirus NS1 protein (ab214337, [D/2/D6/B7] clone, Abcam, 1:400), FOXG1 (ab214337, Abcam, 1:500), Chikungunya virus native protein (MA5-18181, A54Q clone, Invitrogen, 1:50), Usutu virus native protein (MA5-18281, F50F clone, Invitrogen, 1:50), SOX2 (ab5603, Millipore, 1:400), TUBA (mca77g, Bio-Rad, 1:500), SOX1 (4194, Cell signaling, 1:200), pHH3 (06-570, Millipore, 1:500), cleaved CASP3 (ab3623, Millipore, 1:200) diluted in antibody solution [3% fetal bovine serum, 0.2% (vol/vol) Triton X-100 in PBS Ca2+/Mg2+1X] at 4°C overnight. The next day, cells were washed with PBSX three times for 3 minutes and then incubated for 1h at RT with Alexa Fluor®

secondary antibodies and DAPI (D1306, Invitrogen, 1 μg/ml) diluted in antibody solution. After two washes of 3 minutes with PBSX and one wash of 3 minutes with PBS Ca2+/Mg2+, the fixed cells were mounted on microscope slides to perform confocal analysis. All images were acquired using a laser scanning confocal microscope (Nikon, Eclipse Ti) or a laser scanning confocal microscope Zeiss LSM 9.10 (Carl Zeiss).

Measurement of the nuclear-cytoplasmatic percentage of fluorescence

Using ImageJ software (https://imagej.nih.gov/ij/), a ROI (Region Of Interest) was drawn to select the nucleus and the total cell area (cytoplasm and nucleus) of each cell and to measure the intensity of fluorescence in each selection in the channel of interest. Moreover, one small circle was selected out of each cell to measure the background fluorescence. The following parameters were measured:

- ROI Area: the number of pixels into the ROI
- Mean Gray Value: $\frac{\Sigma\, gray\ intensity\ of\ pixels\ into\ the\ ROI}{number\ of\ pixels\ into\ the\ ROI}$
- Integrated Density: $ROI\ Area \times Mean\ Gray\ Value$

Then, the Corrected Total Cellular Fluorescence (CTCF) was calculated:

- $CTCF_{total} = IntDen_{total} - (ROI\ Area_{total}\ x\ Mean\ Gray\ Value_{backgroud})$
- $CTCF_{nuclear} = IntDen_{nuclear} - (ROI\ Area_{nuclear}\ x\ Mean\ Gray\ Value_{backgroud})$
- $Nuclear\ fluorescence\ \% = \frac{CTCF_{nuclear}}{CTCF_{total}}\ x\ 100$

Once obtained these values, the target marker nuclear fluorescence of mock and infected cells was compared.

Reverse-transcriptase quantitative PCR (RT-qPCR)

hiPS-NPCs were cultured in a 6-wells cell culture multi-well plate and infected with $ZIKV^{Br.}$ strain with MOI=1 until DPI 3. Total RNA was extracted using the RNeasy Plus mini kit (74134, Qiagen) as per manufacturer instructions. cDNA synthesis was performed using 1 μg of total RNA as the template and reverse transcribed using GoScript™ Reverse Transcriptase (A5001, Promega) primed with 0.50 μg of random hexamers. RT-qPCR was performed using the QuantStudio™ 3 Real-Time PCR System (Applied Biosystems™, A28137) and the SensiMixTM SYBR®No-ROX kit (Meridian BIOSCENCE, QT650-05) as per the manufacturer's protocol. The forward and reverse primers were (5' to 3'): TGGACGCAGACCTTGAGAAC and GGGCACCTTTACTACGAATGC for *FOXG1*, GCGGAGGAGAACAAAC AGATC and GAGGGCGGATTGGAAATGAAC for *CCND1*, CTCTCAGGGTCGAAAACGGC and GCGGATTAGGGCTTCCTCTTG for *CDKN1A*, CGCAGGAATAAGGAAGCGACC and GGCATTTGGGGAACCGTCTG for *CDKN1B*, and AGCTGAACGGGAAGCTCACT and AGGTCCACCACTGACACGTTG for *GAPDH*. RT-qPCR was performed in 20 µl with the following parameters: an activation step of 95°C for 10 min, followed by 40 cycles of 95°C for 10 sec, 60°C for 15 sec, and 72°C for 15 sec, and a melt curve step of 95°C for 15 sec, 60°C for 1 min, and 95°C for 15 sec. The generation of specific PCR products was confirmed by melting curve analysis. The data were analyzed using the $2^{-\Delta\Delta Ct}$ method with all samples normalized to *GAPDH* and mock condition.

Western blotting

hiPS-NPCs were cultured in a 6-well multi-well plate and infected with $ZIKV^{Br.}$ strain with MOI=1 until DPI 3. Then, cells were lysed in ice with RIPA buffer (R0278, Sigma) with 1% SDS, protease inhibitor 7x (11836170001, Sigma), and phosphatase inhibitor 10x (04906, Roche). They were quantified with DC protein assay (500-0116, Bio-Rad), and boiled at 70°C for 10 min with Laemmli 4x (161-0747, Bio-Rad). Next, lysates were run in Mini-PROTEAN precast gel (4568023, Bio-Rad) in Running buffer at 300V for 15 min and transferred on PVDF membrane (03010040001, Sigma) with Trans-Blot Turbo System (Bio-Rad) at 2.5A for 7 min. The membrane was washed with TBST (0.05% Tween v/v in TBS) for 5 min in rocking plate at RT, incubated with Blocking solution (Milk 5% w/v in TBST) for 1 h in rocking plate at RT, washed with TBST for 2 min in rocking plate at RT, and finally incubated with primary antibodies FOXG1 (ab214337, Abcam, 1:2000), TUBA (3873, Cell signaling, 1:8000), and NS3 (133309, GeneTex, 1:2000) diluted in antibody solution (Milk 1% w/v in TBST) at 4°C overnight in rocking plate. The next day, the membrane was washed 3 times with TBST for 10 min in rocking plate at RT and incubated with secondary antibodies conjugated to peroxidase (SAB3700934, Sigma, 1:2000; SAB3701105, Sigma, 1:2000) diluted in antibody solution for 1h at RT in rocking plate. Finally, after 3 x 10 min-washes in TBST, the membrane was revealed using ECL substrates (170-5060, Bio-Rad) with Chemidoc system. Expected size of western blotting bands: 70 kDa for FOXG1, 69 kDa for NS3, and 50 kDa for TUBA.

Determination of cell death

A549 cells on 24-well plates were stained live with 1 µg/ml Hoechst (33258, Sigma) and 5 µM propidium iodide (PI) (P4170, Sigma) dissolved in culture medium as previously described (5). Nuclear morphology was assessed using an EVOS FLoid Imaging System (Thermofisher, 4471136) inverted microscope with 20X, 0.43 NA phase-contrast objective using the appropriate filter set for Hoechst, PI, GFP and a charge-coupled device camera. The number of PI-positive cells was expressed as a percentage of total cells in the field. Resultant images were processed using Image J software analysis (Wayne Rasband, National Institutes of Health, Bethesda, MD).

References

1. Sousa AMM, Zhu Y, Raghanti MA, Kitchen RR, Onorati M, Tebbenkamp ATN, et al. Molecular and cellular reorganization of neural circuits in the human lineage. Science [Internet]. 2017 Nov 24;358(6366):1027–32. Available from: https://www.sciencemag.org/lookup/doi/10.1126/science.aan3456
2. Dell'Anno MT, Wang X, Onorati M, Li M, Talpo F, Sekine Y, et al. Human neuroepithelial stem cell regional specificity enables spinal cord repair through a relay circuit. Nature Communications. 2018;9(1):3419.
3. Onorati M, Li Z, Liu F, Sousa AMM, Nakagawa N, Li M, et al. Zika Virus Disrupts Phospho-TBK1 Localization and Mitosis in Human Neuroepithelial Stem Cells and Radial Glia. Cell Reports. 2016;16(10):2576–92.
4. Lai M, Iacono E, Spezia PG, Lottini G, La Rocca V, Quaranta P, et al. A low-cost simple test for weekly detection of Mycoplasma hyorhinis and arginini contaminations in cell cultures and viral preparations. Journal of Virological Methods. 2022 Jan 1;299:114327.
5. D'Orsi B, Engel T, Pfeiffer S, Nandi S, Kaufmann T, Henshall DC, et al. Bok Is Not Pro-Apoptotic But Suppresses Poly ADP-Ribose Polymerase-Dependent Cell Death Pathways and Protects against Excitotoxic and Seizure-Induced Neuronal Injury. Journal of Neuroscience [Internet]. 2016 Apr 20 [cited 2021 Dec 9];36(16):4564–78. Available from: https://www.jneurosci.org/lookup/doi/10.1523/JNEUROSCI.3780-15.2016
