# Supplementary material for: Zika virus induces FOXG1 nuclear displacement and downregulation in human neural progenitors
Source: Stem Cell Reports. 2022 Jun 16;17(7):1683–98. doi: 10.1016/j.stemcr.2022.05.008 (PMC9287670; doi:10.1016/j.stemcr.2022.05.008)
Supplement: Document S2. Article plus supplemental information [file mmc2.pdf]

Stem Cell Reports
Article
OPEN ACCESS

Zika virus induces FOXG1 nuclear displacement and downregulation in human neural progenitors

Giulia Lottini,[1,2,7] Matteo Baggiani,[3,7] Giulia Chesi,[1,7] Beatrice D'Orsi,[4,5] Paola Quaranta,[1] Michele Lai,[1] Laura Pancrazi,[4] Marco Onorati,[3,8] Mauro Pistello,[1,8] Giulia Freer,[1,8] and Mario Costa[4,5,6,8,*]
[1]Centro Retrovirus, Department of Translational Research, University of Pisa, Pisa 56127, Italy
[2]Department of Medical Biotechnologies, University of Siena, Siena 53100, Italy
[3]Unit of Cell and Developmental Biology, Department of Biology, University of Pisa, Pisa 56127, Italy
[4]Institute of Neuroscience, Italian National Research Council (CNR), Via Moruzzi, 1, Pisa 56124, Italy
[5]Centro Pisano ricerca e implementazione clinica Flash Radiotherapy (CPFR@CISUP), Presidio S. Chiara, ed.18 via Roma, 67, Pisa 56126, Italy
[6]Laboratory of Biology "Bio@SNS", Scuola Normale Superiore, Piazza dei Cavalieri, Pisa 56124, Italy
[7]These authors contributed equally
[8]Co-last authors
*Correspondence: costa@in.cnr.it
https://doi.org/10.1016/j.stemcr.2022.05.008

SUMMARY

Congenital alterations in the levels of the transcription factor Forkhead box g1 (*FOXG1)* coding gene trigger "FOXG1 syndrome," a spectrum that recapitulates birth defects found in the "congenital Zika syndrome," such as microcephaly and other neurodevelopmental conditions. Here, we report that Zika virus (ZIKV) infection alters FOXG1 nuclear localization and causes its downregulation, thus impairing expression of genes involved in cell replication and apoptosis in several cell models, including human neural progenitor cells. Growth factors, such as EGF and FGF2, and Thr271 residue located in FOXG1 AKT domain, take part in the nuclear displacement and apoptosis protection, respectively. Finally, by progressive deletion of FOXG1 sequence, we identify the C-terminus and the residues 428–481 as critical domains. Collectively, our data suggest a causal mechanism by which ZIKV affects FOXG1, its target genes, cell cycle progression, and survival of human neural progenitors, thus contributing to microcephaly.

INTRODUCTION

Zika virus (ZIKV) is a positive-sense single-stranded RNA (ssRNA$^+$) virus first described in 1947 in Uganda and transmitted by *Aedes* mosquito or from mother to fetus (Musso and Gubler, 2016). Up to 2013, the African and Asian lineages of ZIKV caused no significant pathogenicity in humans; conversely, in 2015, the incidence of microcephaly in infants born to ZIKV-infected mothers significantly increased in Brazil and other countries (Butler, 2016; Faria et al., 2016; Heymann et al., 2016). A large percentage of ZIKV-infected adults develop mild symptoms, resembling those caused by other Arboviruses. Nevertheless, ZIKV causes congenital brain malformations in the fetus, including microcephaly (Vasudevan et al., 2018). Indeed, in animal models, as well as in cell culture and organoids, ZIKV preferentially infects neural stem cells (NSCs) that exhibit a degree of susceptibility inversely proportional to their differentiation state (Baggiani et al., 2020). However, little is known about the mechanistic link between infection and microcephaly.

Notably, ZIKV causes brain development impairment that resembles the congenital alterations at the anatomic, symptomatological, or molecular levels induced by mutations/downregulation of *FOXG1* (Boggio et al., 2016; Cargnin et al., 2018; Florian et al., 2012; VHP et al., 2020). FOXG1 is an evolutionarily conserved transcription factor of 481 amino acids (aa) in mice and 489 aa in humans (Kaestner et al., 2000). In vertebrates, proper *FoxG1* expression is essential for telencephalon development and, during early phases of corticogenesis, it orchestrates forebrain development, including cortical expansion, NSC self-renewal, and cell commitment (Kumamoto and Hanashima, 2017). Deregulation or mutations in *FOXG1* have been identified in many pathological conditions, including FOXG1 syndrome, autism spectrum disorders, and several types of cancers (Bulstrode et al., 2017; Mariani et al., 2015; Mitter et al., 2018).

Although FOXG1 is predominantly nuclear, its subcellular localization is controlled post-translationally by different stimuli, including growth factors (GFs), such as fibroblast growth factor-2 (FGF2) and insulin-like growth factor 1 (IGF-1), which activate ERK and AKT pathways (Regad et al., 2007). In the nucleus, FOXG1 transcriptionally activates or represses multiple targets, including *Fgf8* (Kumamoto and Hanashima, 2017; Zhao et al., 2021), *Ccnd1* (*Cyclin D1*), and cell-cycle inhibitors such as *Cdkn1a* (*p21*) (de Filippis et al., 2012; Seoane et al., 2004) and *Cdkn1b* (*p27*) (Cargnin et al., 2018). Conversely, in the cytoplasmic compartment, FOXG1 is found in mitochondria, where it coordinates bioenergetics and the early phases of neuronal differentiation (Pancrazi et al., 2015) and apoptosis (Dastidar et al., 2011).

We and others demonstrated that ZIKV alters cell cycle progression, apoptosis, and mitosis, leading to mitochondrial failure, oxidative stress, and DNA damage in cortical

This is an open access article under the CC BY license (http://creativecommons.org/licenses/by/4.0/).

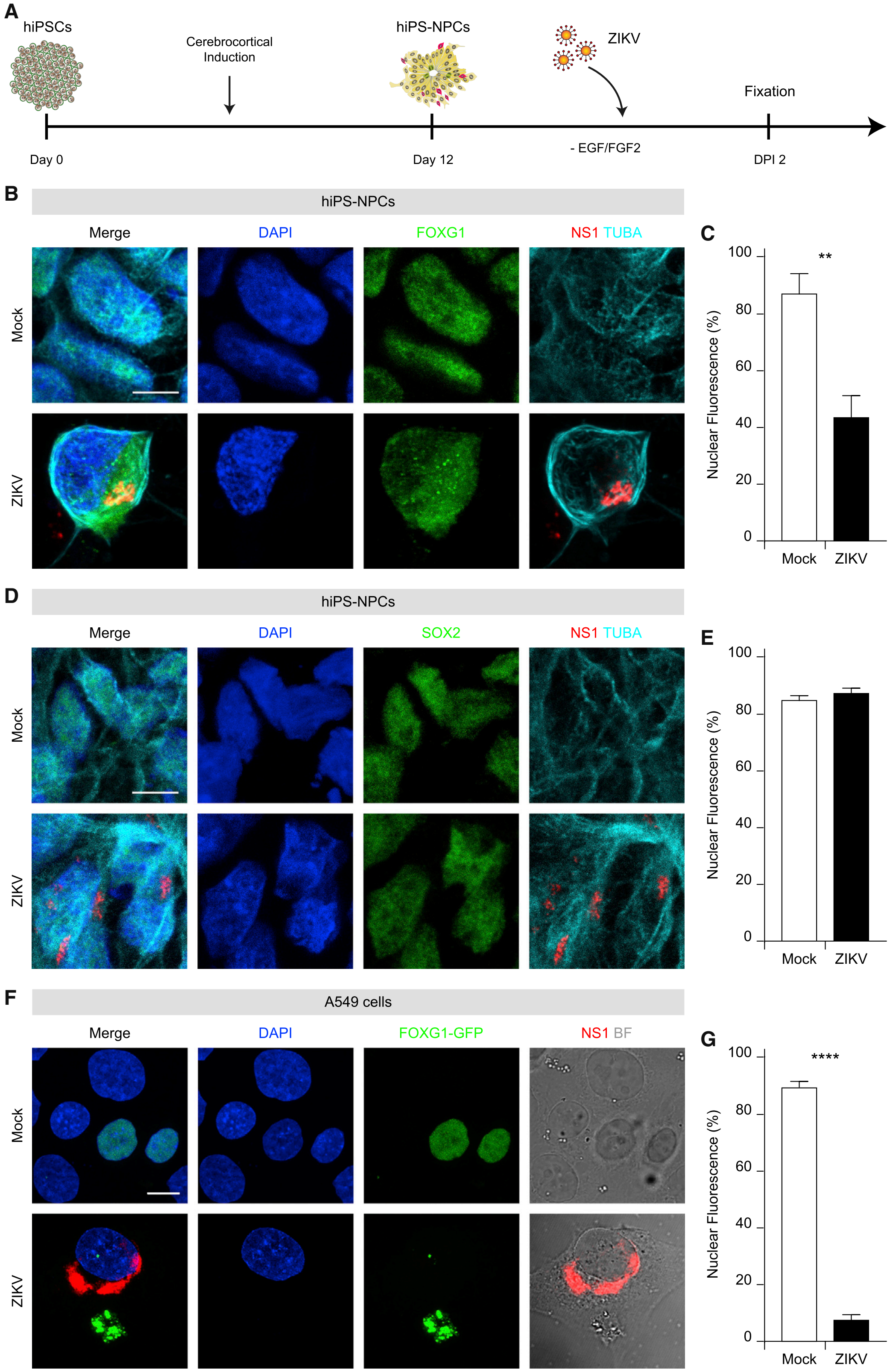

(legend on next page)

neural stem/progenitor cells (Hammack et al., 2019; Ledur et al., 2020; Onorati et al., 2016; Qian et al., 2016; Rothan et al., 2019; Tang et al., 2016; Yang et al., 2020; Zhang et al., 2019). In particular, ZIKV produces supernumerary centrosomes and disrupts pTBK1 localization from centrosomes to mitochondria in human neocortical neuroepithelial stem (NES) cells, leading to mitosis impairment and microcephaly (Onorati et al., 2016).

Here, we hypothesize that a common mechanism might exist between congenital ZIKV infection-caused microcephaly and FOXG1. We investigated FOXG1 localization in telencephalic neural progenitor cells derived from human induced pluripotent stem cells (hiPS-NPCs) and human neocortical NES cells, both expressing endogenous FOXG1, as well as in A549 cells, transiently expressing FOXG1. We demonstrate that (1) FOXG1 is displaced from the nucleus to the cytoplasm and downregulated following ZIKV infection; (2) it is a specific target of ZIKV, but not of other Arboviruses, such as Usutu virus (USUV) and Chikungunya virus (CHIKV); (3) its C-terminal domain is responsible for mediating FOXG1 mislocalization; and (4) FOXG1-targeted genes are altered following ZIKV infection, thus defining a causal link between ZIKV, FOXG1, cortical NSC vulnerability, cell cycle alteration, and cell death.

RESULTS

ZIKV infection produces FOXG1 nuclear displacement

Proper FOXG1 levels are essential for a correct neural progenitor fate since pathological FOXG1 downregulation results in FOXG1 syndrome. Because FOXG1 and congenital Zika syndromes display common clinical traits, we first investigated the impact of ZIKV infection on FOXG1. We obtained NPCs from hiPSCs after cerebro-cortical induction, which recapitulates early stages of human neurodevelopment (Figure 1A). hiPSCs represent an extraordinary *ex vivo* source for the derivation of NPCs and a powerful tool to study human neurodevelopmental diseases (Dell'Amico et al., 2021). In our protocol, we generated hiPS-NPCs with a dorsal telencephalic identity, endowed with a cortical fate (Sousa et al., 2017).

To verify the susceptibility of hiPS-NPCs to ZIKV infection, we monitored them up to 3 days post-infection (DPI) with ZIKV MP1751. The nuclear-to-total ratio of FOXG1 signal was quantified and showed a time-dependent pattern (Figures S1A and S1C). We observed progressive and widespread ZIKV infection by immunolabeling for ZIKV non-structural protein 1 (NS1), with over 63.07% ± 14.30% of infected hiPS-NPCs at DPI 3 (Figures S1A and S1B). In uninfected ("mock") hiPS-NPCs, FOXG1 was mostly localized within the nucleus, as previously reported in normal neuroprogenitors in the developing human telencephalon (Onorati et al., 2014) (Figures 1B, 1C, S1A, and S1C). ZIKV-infected hiPS-NPCs still displayed FOXG1 nuclear localization at DPI 1 (Figures S1A and S1C), whereas it progressively shifted toward the cytosol, with maximum reduction in the nucleus at DPI 2 (Figures S1A and S1C; 85.04% ± 4.04% versus 42.44% ± 4.40%, respectively), with an 8-fold change in the cytosolic/nuclear ratio of FOXG1 compared with mock ($p < 0.05$, one-way ANOVA). Therefore, we defined DPI 2 as the optimal time post-infection for subsequent experiments on hiPS-NPCs.

To test whether the effect of ZIKV infection was specific to FOXG1 or could impinge also on other transcription factors, we examined the expression of SOX1 and SOX2, which are active in neural development and essential for maintaining NSC self-renewal (Graham et al., 2003; Kan et al., 2007). Our results indicated that ZIKV infection did not affect SOX1 or SOX2 patterns (Figures 1D, 1E, S2A, and S2B).

Despite a modest dissimilarity between human and murine FOXG1 aa sequences is present (481 aa mouse versus

Figure 1. Mislocalization of FOXG1 after ZIKV infection in hiPS-NPCs and A549 cells
(A) Schematic representation of NPC derivation and viral infection.
(B) Representative confocal images of FOXG1, ZIKV NS1, TUBA (TUBA1A, α-tubulin), and DAPI in mock and ZIKV-infected hiPS-NPCs. Analyses were performed at DPI 2. Scale bar, 10 μm.
(C) Bar plot indicating the ratio of FOXG1 nuclear fluorescence on total fluorescence in mock and ZIKV-infected conditions (total cells, n = 40), $p < 0.01$.
(D) Representative confocal images of mock and ZIKV-infected hiPS-NPCs showing SOX2 pattern after ZIKV infection at DPI 2. Scale bar, 10 μm.
(E) Bar plot indicating the ratio of SOX2 nuclear fluorescence on total fluorescence in mock and ZIKV-infected conditions (total cells, n = 40), $p > 0.05$.
(F) Representative confocal images of FOXG1-GFP transfected A549 cells. BF, Bright field. Analyses were performed at DPI 1. Scale bar, 10 μm.
(G) Bar plot indicating the ratio of FOXG1 nuclear fluorescence on total fluorescence in mock and ZIKV-infected conditions. Data are shown as mean ± SEM (total cells, n = 37), $p < 0.0001$ (Kolmogorov-Smirnov test). (C and E) Data are shown as mean ± SD (unpaired Student's t test). See also Figures S1 and S2.

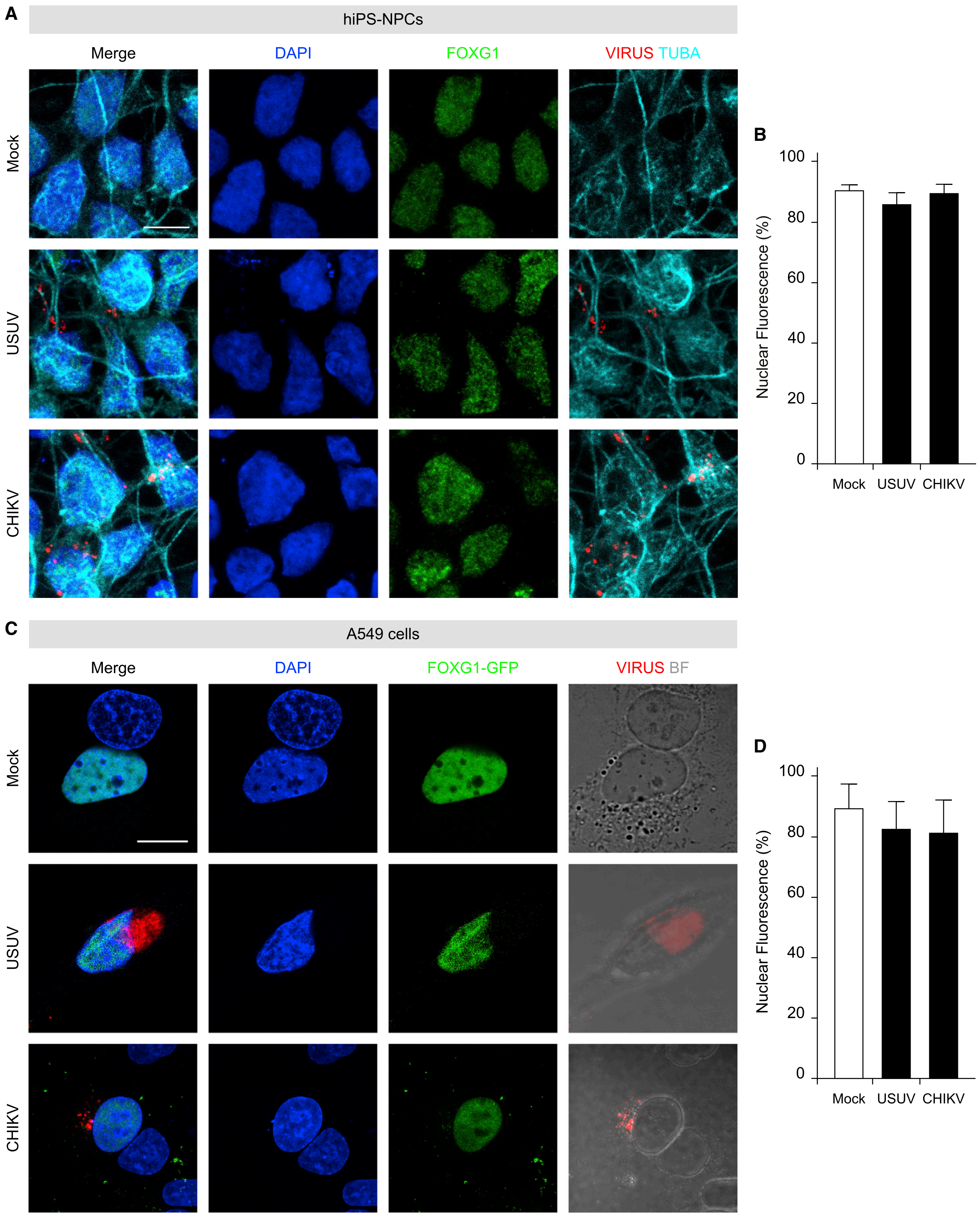

(legend on next page)

489 aa human), we used murine FOXG1 constructs fused to GFP at its C-terminus (FOXG1-GFP) and transiently transfected them in A549 cells and, 24 h later, infected them with ZIKV. Notably, in mock conditions, FOXG1-GFP localized to the nucleus, while it was displaced to the cytoplasm after ZIKV infection at DPI 1 (Figures 1F and 1G). Altogether, these findings show that ZIKV-induced FOXG1 nuclear displacement occurs both in hiPS-NPCs endogenously expressing FOXG1, as well as in exogenous FOXG1-expressing A549 cells. Next, to evaluate whether FOXG1 relocation was specific for ZIKV, both hiPS-NPCs and A549 cells, transiently expressing FOXG1-GFP, were infected with two different Arboviruses: USUV, belonging to the same genus as ZIKV, or the Asian strain of CHIKV, belonging to the Togaviridae family. Interestingly, we did not detect significant changes in FOXG1 localization after either USUV or CHIKV infection in hiPS-NPCs (Figures 2A and 2B) or in A549 cells (Figures 2C and 2D). These data reinforce the finding that ZIKV, but not other viruses, specifically perturbs FOXG1 nuclear pattern.

Growth factors prevent FOXG1 displacement following ZIKV infection

To further explore FOXG1 nuclear pattern disruption, we turned to NES cells, a model of human NSCs with neocortical identity, where the effect of ZIKV infection has been examined in detail (Onorati et al., 2016). NES cells are neurogenic, tripotent, and positive for neuroprogenitor markers, such as SOX1 and SOX2. Remarkably, they retain positional identity as confirmed by the expression of regional markers typical of the area they are derived from, including FOXG1. Unexpectedly, we could not detect any evident nuclear/total fluorescence ratio alteration of FOXG1 in ZIKV-infected NES cells (Figure S3).

To explain this result, we hypothesized that the different culture conditions between hiPS-NPCs and NES cells could affect FOXG1 shuttling. Indeed, while NES cells are exposed to EGF and FGF2 to propel their self-renewal state (Onorati et al., 2016), hiPS-NPCs are maintained into a neural medium devoid of GFs. For this reason, we exposed hiPS-NPCs to EGF and FGF2 for 13 days, after which cells were infected with ZIKV in the presence of both GFs (Figure 3A). Similar to NES cells, no changes in FOXG1 nuclear localization were observed in this condition (Figures 3B and 3C). Next, to evaluate the individual contribution of EGF and FGF2, we separately exposed hiPS-NPCs to each factor and found that EGF and/or FGF2 maintained FOXG1 nuclear localization following ZIKV infection (Figure 3C).

In parallel, we evaluated whether treatment with the same cocktail of GFs exerted comparable effects in ZIKV-infected A549 cells expressing FOXG1-GFP. Again, EGF and FGF2 precluded FOXG1 relocation following ZIKV infection (Figures 3D and 3E).

Several reports in the literature show FGF2 level association with infection by ZIKV (Limonta et al., 2019). To investigate the potential involvement of FGF2 in our experimental paradigm, we performed RT-qPCR to monitor *FGF2* gene expression. Progressive increase, from DPI 1 to 3, was observed following ZIKV infection (Figure S4A). Furthermore, in the attempt to “revert” the disruption of FOXG1 nuclear localization by ZIKV infection, we exposed hiPSC-NPCs to GFs after ZIKV infection and showed that they preserved FOXG1 nuclear pattern (Figures S4B and S4C). Altogether, these data suggest that the impact of ZIKV infection on FOXG1 is modulated by the presence of EGF and/or FGF2, in our experimental paradigm.

Thr271 in FOXG1 AKT domain is involved in ZIKV-induced FOXG1 nuclear displacement and apoptosis protection

Given that GFs prevented the effect of ZIKV infection on FOXG1 nuclear displacement and AKT signaling is pivotal for FOXG1 functional activation and intracellular localization (Baek et al., 2015; Dastidar et al., 2011), we molecularly dissected the activity of FOXG1 domains. First, we studied whether the putative FOXG1 AKT domain (aa 266–271 RXRXXS*/T*X) and, specifically, threonine 271 (Thr271) was involved in ZIKV-induced nuclear displacement (Hettige and Ernst, 2019; Regad et al., 2007) and in the previously suggested role in apoptosis (Dastidar et al., 2011). For this reason, we generated phospho-mimetic and phospho-defective mutants of FOXG1, fused to GFP at their C-terminal domain, where Thr271 was substituted with a non-phosphorylatable aspartic acid (T271D) or alanine (T271A), respectively (Figure S5B), and examined their

Figure 2. Other arboviruses do not affect FOXG1 localization in hiPS-NPCs and A549 cells

(A) Representative confocal images of FOXG1, Virus, TUBA (α-tubulin), and DAPI of mock and hiPS-NPCs infected with USUV or CHIKV. Analyses were performed at DPI 2. Scale bar, 10 μm.

(B) Bar plot indicating the ratio of FOXG1 nuclear fluorescence on total fluorescence in mock and infected conditions (total cells, n = 60), $p > 0.05$.

(C) Representative confocal images of FOXG1-GFP transfected A549 cells after infection with USUV or CHIKV. BF, Bright field. Analyses were performed at DPI 1. Scale bar, 10 μm.

(D) Bar plot indicating the ratio of FOXG1 nuclear fluorescence on total cellular fluorescence in mock and infected conditions (total cells, n = 36), $p > 0.05$. (B and D) Data are shown as mean ± SD (one-way ANOVA, post hoc Tukey’s test).

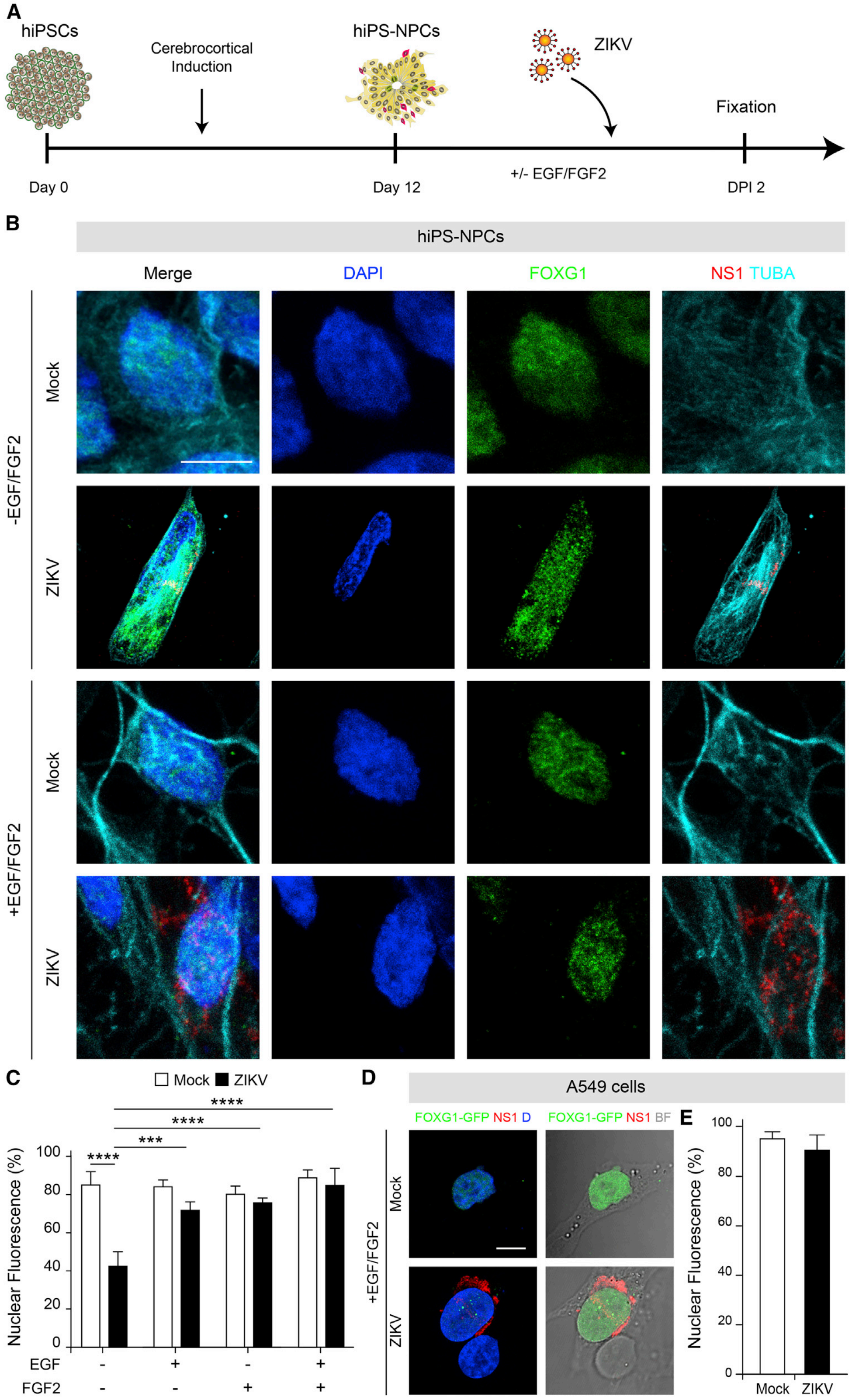

(legend on next page)

effects on FOXG1 localization and potential role in apoptosis in A549 cells (Figure 4A). Both mutants displayed a similar nuclear/cytoplasmatic ratio, typical of wild-type (WT) FOXG1, in A549 mock and infected cells (Figures 4A, 4B, 1F, and 1G).

To confirm that the phospho-mutants of FOXG1 retained functional activity, we tested whether the known anti-apoptotic action of FOXG1 was still present after T271 mutation. We transiently expressed T271D and T271A mutants, along with WT FOXG1-GFP and GFP only, in A549 cells. The day after transfection, cells were exposed to the apoptosis-inducing protein kinase inhibitor Staurosporine (STS) and cell injury was quantified 24 h later by propidium iodide (PI) uptake and Hoechst 33258 staining of nuclear chromatin (Figure 4C) (D'Orsi et al., 2016). Strikingly, phospho-defective T271A failed to afford protection against STS-induced apoptosis, while the effect of the phospho-mimetic form T271D did not differ from WT FOXG1 (Figure 4C), suggesting that Thr271 in FOXG1 is critical for the proper balance of survival/apoptosis.

FOXG1 aa 428–481 are responsible for mediating ZIKV effects

To investigate which other FOXG1 regions, in addition to Thr271, contributed to the nuclear displacement in response to ZIKV, we used FOXG1-GFP fusion peptides (Pancrazi et al., 2015) and generated further progressive deletions of FOXG1 at its N- and C-termini (Figures S5C–S5F). In mock A549 cells, the intracellular distribution of N- (aa 1–171) and C- (aa 315–481) FOXG1-GFP fusion peptides, lacking the DNA binding domains in the Forkhead Domain (FHD), resulted diffused in both the nucleus and cytoplasm (Figures 5A and 5C) (Hanashima et al., 2002). Following ZIKV infection, C-FOXG1-GFP (aa 315–481 and aa 428–481) showed significant discrete cytoplasmic clusters (Figures 5C and 5D). In contrast, intracellular localization of N-FOXG1-GFP (aa 1–171) was identical in both mock and ZIKV-infected cells (Figures 5A and 5B). Next, to evaluate the role of the central region of FOXG1 (aa 234–391) containing Thr271 but lacking the N- and C-termini, we transfected A549 cells with FOXG1-GFP (aa 234–391; Figure 5A). In this context, A549 cells displayed a fluorescence pattern that did not change following ZIKV infection. These results suggest that other FOXG1 domains located at the C-terminal domains contributed to nuclear displacement (Figure 5B).

To exclude possible differences that may result from the N-terminal dissimilarity between mouse and human FOXG1 (Figure S5G), we fragmented human FOXG1 in two constructs generating hN-FOXG1 (aa 1–280) and hC-FOXG1 (aa 280–489) peptide fused to GFP (Figure S6A). Consistently with the results with mouse FOXG1 fusion peptides, intracellular distribution of hC-FOXG1-GFP, lacking the FHD, was diffused in both nuclear and cytoplasmic areas (Figure S6B); however, following ZIKV infection, discrete clusters became evident in the cytoplasm (Figure 5D). Conversely, intracellular localization of hN-FOXG1-GFP, presenting the FHD, was predominantly nuclear in both mock and ZIKV-infected cells (Figures 5B and S6B).

In conclusion, we identified the FOXG1 C-terminus as a critical region mediating the effect of ZIKV infection and we narrowed down a minimal region of 53 aa (428–481), located at the extreme C-terminus, as an important stretch modulating FOXG1 relocation after ZIKV infection.

Brazilian ZIKV infection induces FOXG1 nuclear displacement and downregulation, dysregulation in FOXG1 downstream genes, and impacts on cell cycle progression and survival

Because ZIKV-related microcephaly was generally found in patients infected by the Brazilian strain of ZIKV (ZIKV$^{Br.}$) during the 2015 outbreak, we investigated whether ZIKV$^{Br.}$ had similar effects on FOXG1 as the Uganda strain used thus far (Figure 6A). hiPS-NPCs were infected with the Brazil/2016/INMI1 ZIKV strain that induced, at DPI 2 and 3, significant FOXG1 nuclear displacement (Figures 6B and 6C) and considerable decrease in FOXG1 total fluorescence at DPI 3 (Figure 6D). We then performed RT-qPCR, confirming transcriptional reduction of *FOXG1* at DPI 3, but not earlier (Figure 6E). FOXG1 protein decrease was confirmed by Western blot assay at DPI 3 (Figure 6F).

Figure 3. ZIKV-induced FOXG1 displacement is inhibited by GF treatment in hiPS-NPCs and A549 cells
(A) Schematic representation of NPC derivation from hiPSCs and viral infection in the presence of EGF and FGF2 (GFs).
(B) Representative confocal images of FOXG1, ZIKV NS1, TUBA (α-tubulin), and DAPI in mock and ZIKV-infected hiPS-NPCs in the absence and in the presence of GFs. Analyses were performed at DPI 2. Scale bar, 10 μm.
(C) Bar plot indicating the ratio of FOXG1 nuclear fluorescence on total fluorescence in mock and ZIKV-infected conditions. Data are shown as mean ± SD (total cells, n = 160), p < 0.001 (two-way ANOVA, post hoc Tukey's test).
(D) Representative confocal images of FOXG1-GFP transfected A549 cells in the presence of GFs, infected or not with ZIKV. BF, Bright field; D, DAPI. Analyses were performed at DPI 1. Scale bar, 10 μm.
(E) Bar plot indicating the ratio of FOXG1 nuclear fluorescence on total fluorescence in mock and ZIKV-infected cells. Data are shown as mean ± SD (total cells, n = 20), p > 0.05 (unpaired Student's t test with Welch's correction). See also Figures S3 and S4.

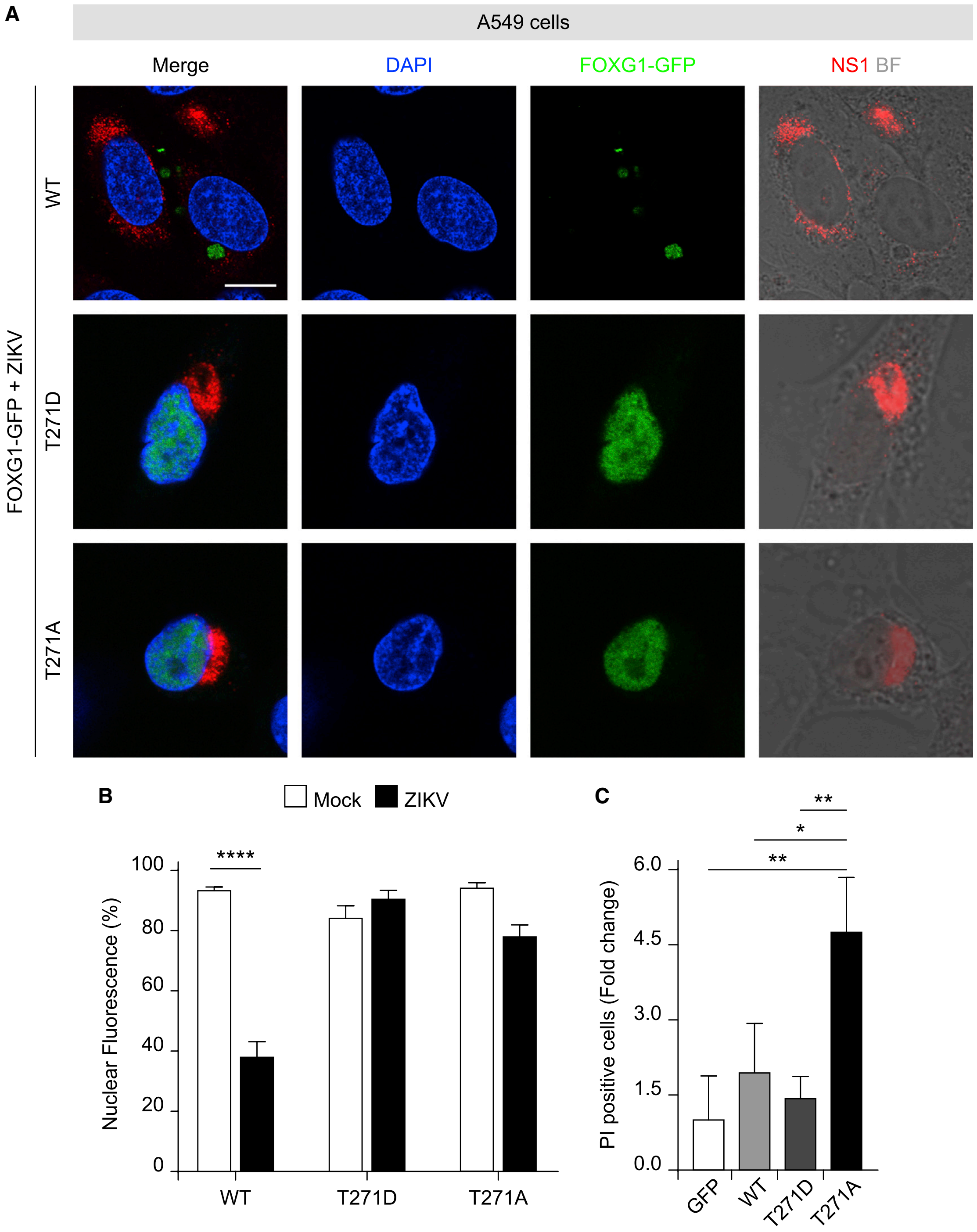

Figure 4. Thr271 in FOXG1 AKT domain is essential for ZIKV-induced FOXG1 nuclear displacement and cell death protection

(A) Representative confocal images of WT FOXG1-GFP, FOXG1-GFP-T271D, and FOXG1-GFP-T271A transfected A549 cells and infected with ZIKV. BF, Bright field. Analyses were performed at DPI 1. Scale bar, 10 μm.

(B) Bar plot indicating the ratio of FOXG1 nuclear fluorescence on total fluorescence in mock and ZIKV-infected conditions (total cells, n = 49), $p < 0.0001$.

(C) A549 cells, transfected with a GFP-only plasmid, WT FOXG1-GFP, FOXG1-GFP-T271D, or FOXG1-GFP-T271A constructs, were treated with Staurosporine (STS) or DMSO after which they were allowed to recover for 24 h. Cell death was assessed by Hoechst

(legend continued on next page)

To evaluate the effects of FOXG1 displacement/reduction following ZIKV$^{Br.}$ infection, we explored timeline expression of several known FOXG1 target genes, focusing on cell replication and apoptosis (Cargnin et al., 2018; Kumamoto and Hanashima, 2017; Seoane et al., 2004; Zhao et al., 2021). We verified the expression of genes involved in the p53-dependent cell-cycle arrest, including *CDKN1A* (*p21*) and *CDKN1B* (*p27*), and *CCND1* (*Cyclin D1*), in mock and ZIKV$^{Br.}$-infected hiPS-NPCs. *CDKN1A* and *CDKN1B* were up-regulated, while *CCND1* was down-regulated in ZIKV$^{Br.}$-infected hiPS-NPCs at DPI 3 (Figures 6G–6I), implying a possibly negative effect of ZIKV$^{Br.}$ on cell cycle progression, i.e. decrease in mitotic index, and activation of apoptosis, as a consequence of p53-dependent cell-cycle arrest (Xiong et al., 2020). Consistently with the previous data, a temporal quantification of hiPS-NPCs by immunofluorescence with markers of proliferation and apoptosis indicated that ZIKV$^{Br.}$ infection caused significant decrease in phosphorylated histone H3 (pHH3; Figure 6J) and substantial increase in cleaved caspase 3 (cCASP3; Figure 6K) at DPI 3, but not at earlier time points. Collectively, these results suggest a link between ZIKV infection, early dysregulation of FOXG1 and its target genes, FOXG1-dependent cell-cycle arrest, and apoptosis in human neural progenitors. The convergent effects result in depletion of the neural progenitor pool, possibly causing developmental alterations observed in congenital Zika syndrome.

DISCUSSION

The severe effects of ZIKV on brain development and the recently demonstrated long-term consequences of perinatal infection emphasize the need to understand the underlying cellular and molecular mechanisms (Blackmon et al., 2020).

Several studies demonstrate the pleiotropic activity of FOXG1 in regulating key cellular functions during embryogenesis (Hou et al., 2020; Kumamoto and Hanashima, 2017). However, no information is available on the effects of viral insults on FOXG1 regulatory activities.

In this report, we identified FOXG1 as a ZIKV target/effector in human neural progenitors. ZIKV infection specifically caused FOXG1 nuclear displacement and downregulation, and alteration of FOXG1 downstream genes involved in cell replication and cell-cycle arrest. Remarkably, no effects were observed on other transcription factors, such as SOX1 and SOX2.

Moreover, using different FOXG1-GFP constructs, we demonstrated that EGF and FGF2, Thr271 (located in the FOXG1 AKT domain; aa 266–271), and the C-terminus of FOXG1 play a key role in modulating ZIKV effects on FOXG1 nuclear localization.

The evidence reported is supported by the notions that ZIKV and FOXG1 may affect the same pathophysiological pathways during fetal development and adulthood. During neurodevelopment, the optimal dosage of FOXG1 is essential to keep telencephalic NPCs in a proliferative state and to prevent apoptosis (Wong et al., 2019). Consistently, dysregulation of FOXG1 results in FOXG1 syndrome, characterized by microcephaly and pathological features overlapping with congenital Zika syndrome (Focosi et al., 2016; Wong et al., 2019). Based on these observations, we investigated the effects of ZIKV infection on FOXG1 intracellular localization and levels in human NPCs obtained from hiPSCs after cerebrocortical induction. To date, ZIKV infection has never been related to changes in transcription factor intracellular localization/activity. Our findings indicate that FOXG1 displacement and downregulation caused by ZIKV infection precedes and then affects known FOXG1 downstream genes, such as *CCND1,* involved in cell cycle progression, or *CDKN1A* and *CDKN1B,* involved in p53-dependent cell-cycle arrest. These effects include reduction in the mitotic index and in apoptosis execution in NPCs, as measured by pHH3 and cCASP3 positivity, respectively. This is consistent with a previous report in which ZIKV infection of hiPSC-NPCs resulted in post-transcriptional changes, *FOXG1* downregulation, upregulation of apoptotic signaling, and downregulation of cell-cycle pathways, in agreement with FOXG1 role in preventing apoptosis and maintaining CDKN1A-mediated proliferation (Jiang et al., 2018).

An intriguing aspect that further expands FOXG1 involvement during ZIKV infection is the oncolytic activity of this virus on different brain tumors (Zhu et al., 2017). Abundant expression of FOXG1 is well-documented in several brain cancers, including glioblastoma (Bulstrode et al., 2017; Dali et al., 2018), where ZIKV is explored as an oncolytic therapeutic option. In this scenario, FOXG1 over-expression in brain tumors and the herein demonstrated capability of ZIKV to downregulate FOXG1 and its downstream genes suggest a mechanism for ZIKV oncolytic action.

T271 in the putative AKT domain (aa 266–271) has been reported to have a role in nuclear-cytoplasmic FOXG1

33258 and propidium iodide (PI) staining. PI-positive nuclei were scored as dead cells and normalized on DMSO treatment. Bar blot indicating fold change in the ratio of PI-positive cells on GFP-only plasmid (total cells, n = 1428), $p < 0.05$. (B and C) Data are shown as mean ± SD; (B) two-way ANOVA, post hoc Tukey's test; (C) One-way ANOVA, post hoc Tukey's test. See also Figures S5.

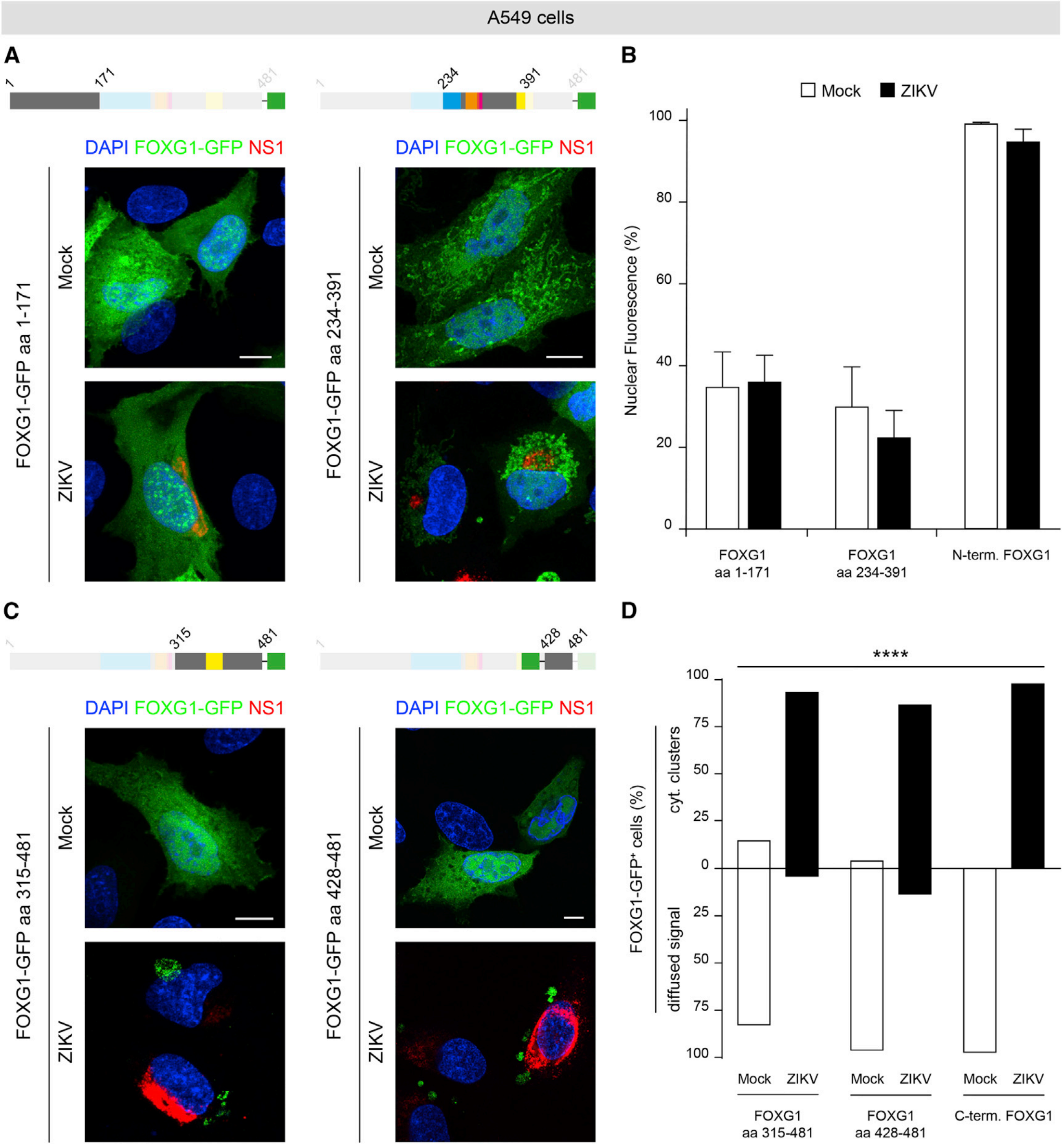

Figure 5. FOXG1 C-terminus is essential for reacting to ZIKV infection

(A) Schematic illustration of FOXG1-GFP constructs. Representative confocal images of FOXG1-GFP aa 1–171 or FOXG1-GFP aa 234–391 transfected A549 cells in mock and ZIKV-infected conditions. Analyses were performed at DPI 1. Scale bar, 10 μm.

(B) Bar plot indicating the ratio of FOXG1 nuclear fluorescence on total fluorescence in mock and ZIKV-infected conditions in mouse FOXG1 aa 1–171, mouse FOXG1 aa 234–391 and human N-terminal FOXG1 aa 1–280 transfected A549 cells. Data are shown as mean ± SD (total cells, n = 29), $p > 0.05$ (two-way ANOVA, post hoc Tukey's test).

(C) Representative confocal images of FOXG1-GFP aa 315–481 or FOXG1-GFP aa 428–481 transfected A549 cells in mock and ZIKV-infected conditions. Analyses were performed at DPI 1. Scale bar, 10 μm.

(D) Bar plot indicating the percentage of cells with FOXG1-GFP diffused signal or FOXG1-GFP cytoplasmic (cyt.) clusters in mock and ZIKV-infected conditions in mouse FOXG1 aa 315–481, mouse FOXG1 aa 428–481, and human C-terminal FOXG1 aa 280–489 transfected A549 cells. Data are shown as mean (total cells, n = 30), $p < 0.0001$ (chi-square test). FHD, Forkhead Domain (blue); MIT, Mitochondrial domain (orange); GTB, GROUCHO/TLE-Binding domain (pink); JBD, JARID1B Binding Domain (yellow); GFP, Green Fluorescence Protein (green). See also Figures S5 and S6.

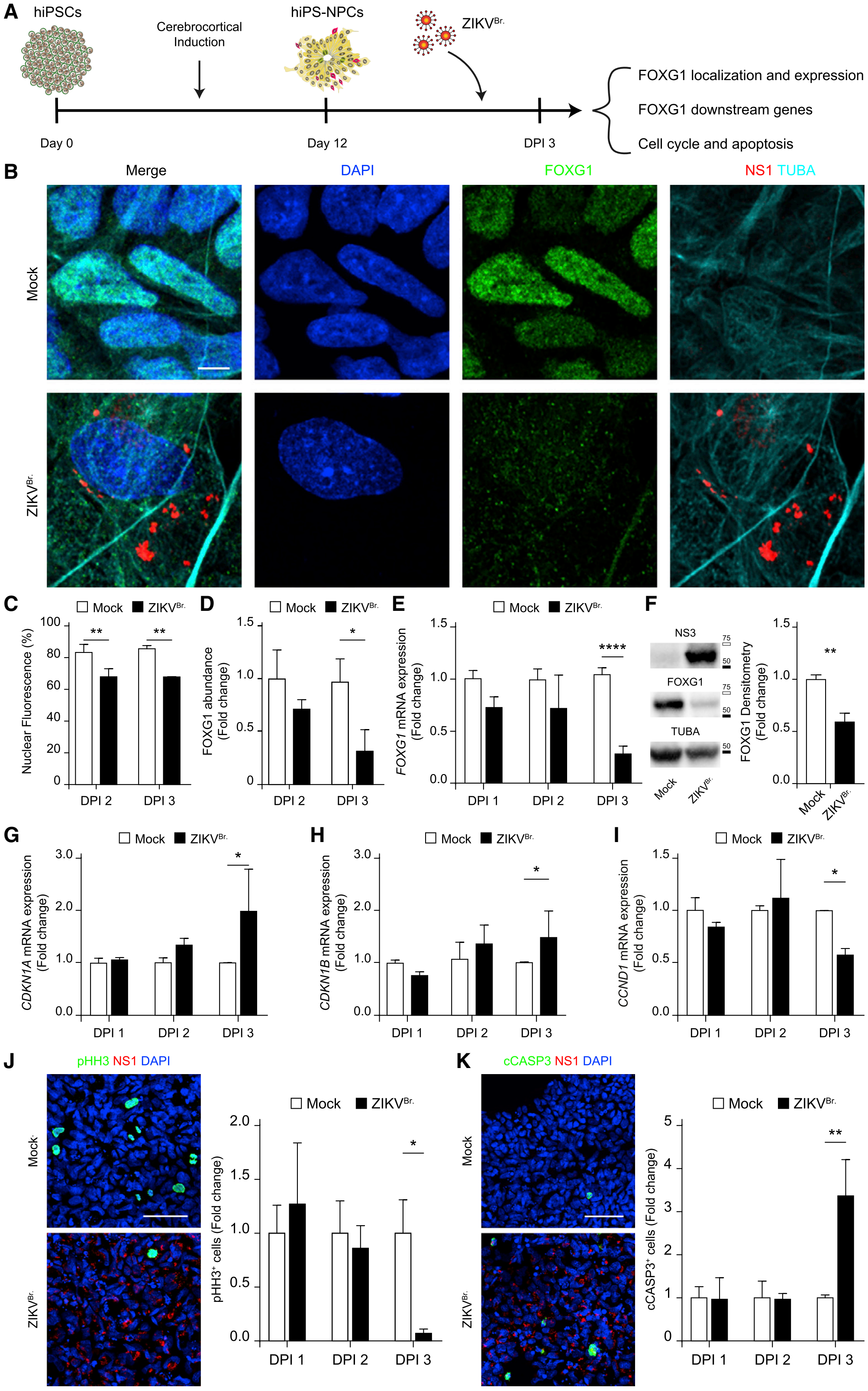

(legend on next page)

mobility and against apoptotic stimuli (Hettige and Ernst, 2019). However, the relationship between GFs, including FGF2, activation signaling of T271, and FOXG1 subcellular localization remains controversial. Regad et al. demonstrated that T-to-A mutation blocks FOXG1 nuclear exit (Regad et al., 2007), whereas Dastidar and colleagues showed that, following IGF-1 treatment, FOXG1 remains in the nucleus, retaining its anti-apoptotic function (Dastidar et al., 2011). In line with the former scenario, our results show that FOXG1 T271 mutants remain in the nucleus, similar to controls, suggesting that T271 is a key residue for FOXG1 mislocalization following ZIKV infection, and plays a critical role in its survival-promoting activity. An implication is that ZIKV infection may interfere, directly or indirectly, with FOXG1 control of the balance among proliferation, differentiation, and apoptosis.

The demonstration that EGF and FGF2 help retain FOXG1 nuclear localization following ZIKV infection, and that ZIKV induces FGF2 expression supports the combinatorial or dose-dependent role of GFs in forebrain shaping, the pathophysiological relevance of FGF2, and suggests a further role of GFs in ZIKV spread. In this context, recent reports also show that ZIKV induces FGF2 expression and FGF2 facilitates virus replication and cell-to-cell spread (Limonta et al., 2019); moreover, in ZIKV-infected pregnant women, blood concentration of FGF2 correlates with the severity of the affected fetuses (Kam et al., 2017) and, finally, FGF2 receptor inhibitors have been suggested as a promising approach for antiviral therapies (Carlin, 2022; Langford et al., 2005; Maddaluno et al., 2020).

Through forced expression of different FOXG1 deletion mutants, previous reports indicate that the first 36 aa at the N-terminal domain are essential for FOXG1 survival-promoting action (Dastidar et al., 2011). Consistently, over-expression of *Foxg1* enhanced the percentage of mitotic cells, while the C-terminus was dispensable (Pancrazi et al., 2015). Using a similar approach, we explored the effect of progressive deletion of FOXG1-GFP to distinguish critical FOXG1 domains responsive to ZIKV infection in A549 cells. This led to the identification of a region of 53 aa (aa 428–481), located at the extreme FOXG1 C-terminus, as a specific site for ZIKV action. Interestingly, once expressed, this mutant, diffused in both nuclear and cytoplasmic areas, showed a cytoplasmic clustered pattern in ZIKV-infected cells. Interestingly, FOXG1 has been shown to possess the ZIKV serine protease (NS2B-NS3) cutting motif (Morazzani et al., 2019). Of note, Li et al. have demonstrated that ZIKV protease hampers neural cell division by degrading Septin-2 (Li et al., 2019).

At the aa level, the C-terminus is a highly conserved portion of FOXG1 where mutations/deletions are found in 15% of FOXG1 syndrome patients (Mitter et al., 2018), suggesting a pivotal role of this domain in regulating FOXG1 functions. Indeed, several FOXG1-networking proteins with key roles in proliferation or repression of transforming growth factor signaling have been identified to directly interact with it (Dali et al., 2018; Marcal et al., 2005; Obendorf et al., 2007; Tan et al., 2003).

In conclusion, our findings identify FOXG1 as a possible pivotal player in ZIKV-associated microcephaly, logically linking viral infection of human neural stem/progenitor cells to FOXG1 relocation and downregulation, cell-cycle arrest, and cell death. Moreover, ZIKV-specific effects on FOXG1, and not on other pan-neural NSC transcription

Figure 6. Brazilian ZIKV infection induces FOXG1 nuclear displacement and downregulation, dysregulation in FOXG1 downstream genes affecting cell cycle progression and survival

(A) Schematic representation of NPC derivation from hiPSCs, viral infection, and effects.

(B) Representative confocal images of FOXG1, Brazilian ZIKV ($ZIKV^{Br.}$) NS1, TUBA (α-tubulin), and DAPI, in mock and $ZIKV^{Br.}$-infected hiPS-NPCs. Analyses were performed at DPI 3. Scale bar, 5 μm.

(C) Bar plot indicating the ratio of FOXG1 nuclear fluorescence on total fluorescence in mock and $ZIKV^{Br.}$-infected conditions (total cells, n = 240), $p < 0.01$.

(D) Bar plot indicating fold change in FOXG1 total fluorescence normalized to mock, in mock and $ZIKV^{Br.}$-infected hiPS-NPCs (total cells, n = 240), $p < 0.05$.

(E) Bar plot indicating fold change in *FOXG1* mRNA level in mock and $ZIKV^{Br.}$-infected conditions (n = 3), $p < 0.0001$.

(F) Western blot and densitometric analysis showed the comparison between the level of FOXG1 in mock and $ZIKV^{Br.}$-infected conditions (n = 4), $p < 0.01$. White box = 75 kDa, Black box = 50 kDa. Bar plot indicating fold change in (G) *CDKN1A* (n = 3), $p < 0.05$; (H) *CDKN1B* (n = 3), $p < 0.05$; and (I) *CCND1* mRNA levels in mock and $ZIKV^{Br.}$-infected conditions (n = 3), $p < 0.05$.

(J) Representative confocal images of pHH3, $ZIKV^{Br.}$ NS1, and DAPI in mock and $ZIKV^{Br.}$-infected hiPS-NPCs at DPI 3. Scale bar, 50 μm. Bar plot indicating fold change in pHH3 positivity normalized to mock, in mock and $ZIKV^{Br.}$-infected hiPS-NPCs (total cells, n = 60.036), $p < 0.05$.

(K) Representative confocal images of cleaved CASP3 (cCASP3), $ZIKV^{Br.}$ NS1, and DAPI in mock and $ZIKV^{Br.}$-infected hiPS-NPCs at DPI 3. Scale bar, 50 μm. Bar plot indicating fold change in cCASP3 positivity normalized to mock, in mock and $ZIKV^{Br.}$-infected hiPS-NPCs (total cells, n = 58.209), $p < 0.01$. (C–K) Data are shown as mean ± SD; (C–E), (G–K) two-way ANOVA, post hoc Tukey's test; (F) unpaired Student's *t* test.

factors, may explain the high vulnerability of telencephalic progenitors during fetal infection. FOXG1 may be the first example of neurodevelopmental transcription factor where localization pattern/level is altered by viral insults. The fact that FOXG1 is a target of ZIKV infection raises the hypothesis that it may serve as a potential mediator for specific external insults that could fine-tune its nuclear localization and functions, eventually resulting in neurodevelopmental disorders.

MATERIALS AND METHODS

Ethical statement

All hiPS and NES cell work was performed according to NIH guidelines for the acquisition and distribution of human tissue for biomedical research purposes and with approval by the Human Investigation Committees and Institutional Ethics Committees of each institute from which samples were obtained. Final approval from the Committee on Bioethics of the University of Pisa was obtained (Review No. 29/2020). De-identified human specimens were provided by the Joint MRC/Wellcome Trust grant (099175/Z/12/Z), Human Developmental Biology Resource (www.hdbr.org). Appropriate informed consent was obtained, and all available non-identifying information was recorded for each specimen. Tissue was handled in accordance with ethical guidelines and regulations for the research use of human brain tissue set forth by the NIH (http://bioethics.od.nih.gov/humantissue.html) and the World Medical Association Declaration of Helsinki (http://www.wma.net/en/30publications/10policies/b3/index.html).

Cell culture

hiPS-NPC, NES, and A549 cell maintenance

Briefly, hiPSCs were dissociated into single cells in StemFlex medium (Thermo Fisher Scientific; #A3349201)] in Matrigel-coated dishes containing 10 μM Y-27632, until confluent, after which dual SMAD inhibition was performed. The medium was changed daily for 12 days and cells were then maintained in a neural differentiation medium. Human NES cells were maintained in NES medium and split, when confluent, once every 5 to 7 days. A549 cells were grown in DMEM high glucose, 1 mM glutamine, 10% fetal calf serum, unless otherwise stated (Lai et al., 2022) (see supplemental information for details).

Viral stocks

The following viral strains were purchased from Public Health England: ZIKV 1308258v, strain MP1751 (Accession number: KY288905.1), CHIKV 0704221v, and USUV 1105081v. All viruses were expanded on Vero cells and titrated as plaque-forming units. ZIKV isolate Brazil/2016/INMI1 (009V-00880) was supplied by the National Institute for Infectious Diseases L. Spallanzani IRCCS.

Transfection and infection

A549 cells were plated onto Lab-Tek chamber slides. The next day, 0.1 μg of the relevant plasmid DNA mixed with 0.5 μL of Lipofectamine (2000) (11668-027; Invitrogen, Italy) was delivered to cells following the manufacturer's instructions. A549, hiPS-NPC, and NES cells were infected at MOI = 1 for 1.5 h in the incubator at 37 °C, 5% CO_2. The virus was then removed and replaced with fresh medium for A549 cells or with one-half conditioning medium and one-half fresh medium for hiPS-NPCs and NES cells.

DNA constructs

All the constructs used in the study have been generated by standard PCR strategy (see supplemental information). The plasmid constructs containing the cDNA coding for the whole mouse FOXG1 fused to the N- and C- termini (GFP-FOXG1 and FOXG1-GFP wt) and 234-391-GFP fragments were previously described (Pancrazi et al., 2015). Plasmids encoding for human FOXG1 fused to GFP was purchased from Origene (Cat: RG207964). Constructs encoding for the N- and C- parts of FOXG1 (1–280 and 280–481 respectively) fused to the GFP at N-terminus, were purchased at IDT, Belgium.

Immunofluorescence

The cells were incubated with primary antibodies (listed in the supplemental information) diluted in antibody solution at 4 °C overnight. Then, Alexa Fluor secondary antibodies and DAPI were diluted in antibody solution and images were acquired using a confocal microscope (see the supplemental information for details).

Reverse-transcriptase quantitative PCR

hiPS-NPCs were infected with $ZIKV^{Br.}$ strain at MOI = 1 until DPI 3. Total RNA was extracted, reverse transcribed, and reverse-transcriptase quantitative PCR (RT-qPCR) was performed. The data were analyzed using the $2^{-\Delta\Delta Ct}$ method with all samples normalized to *GAPDH* and mock condition (see the supplemental information for details).

Western blotting

hiPS-NPCs were infected with $ZIKV^{Br.}$ strain with MOI = 1 until DPI 3. The cells were lysed and the resulting blots were probed with primary antibodies (listed in the supplemental information) in antibody solution at 4 °C overnight. Then, peroxidase-conjugated secondary antibodies were diluted in antibody solution and were detected using enhanced chemiluminescence substrates (170–5060, Bio-Rad) with the Chemidoc system (see the supplemental information for details).

Determination of cell death

A549 cells were treated with Staurosporine 300 nM (37,095, Sigma) or DMSO 1:1000 (D12345, Invitrogen) after which they were allowed to recover for 24 h. Then, they were stained live with 1 μg/mL Hoechst 33258 (Sigma) and 5 μM PI (Sigma). Nuclear morphology was assessed using an inverted microscope. The number of PI-positive cells was expressed as a percentage of total cells in the field.

Statistical analysis

Data are mean ± SD or SEM values from at least three separate experiments after blinded analyses. Differences between groups were

analyzed using appropriate tests as reported for individual figures. Values of all significant correlations are given with degree of significance indicated (*p ≤ 0.05, **p < 0.01, ***p < 0.001, ****p < 0.0001). Total cell number for each experiment is indicated in legends. Data were analyzed with ImageJ software and plotted with GraphPad Prism 7 software (see also the supplemental information).

SUPPLEMENTAL INFORMATION

Supplemental information can be found online at https://doi.org/10.1016/j.stemcr.2022.05.008.

AUTHOR CONTRIBUTIONS

M.O., G.F., M.P., and M.C. designed the study. G.L., M.B., G.C., B.D, P.Q., M.O., G.F., and M.C. designed and performed experiments. L.P. developed the methodology. G.L., M.B., G.C., B.D., and M.L. analyzed the data. G.L., M.B., G.C., B.D., M.O., G.F., and M.C. interpreted the data. G.L., M.B., B.D., M.O., G.F., and M.C. wrote and reviewed the manuscript. M.C., M.O., G.F., and M.P. acquired funding.

ACKNOWLEDGMENTS

This work was supported by DSB.AD011.003 ZIKA and AIRC Individual Grant N: 26044 - IG 2021 to M.C. and partially by Ricerca Finalizzata from Italian Ministry of Health (GR-2018-12367290), NARSAD Young Investigator Grant from the Brain and Behavior Research Foundation (#26565), and 2021 Grant Project from Wings for Life Foundation to M.O.; G.F. and M.P. were partially supported by PRIN (2017KM79NN), H2020-FETOPEN-2018-2020 (862714) and POR FESR Toscana 2014-2020. We thank Dr. Vania Liverani (SNS Pisa), Alfredo Rosellini (Virology Unit, Pisa) for technical support in cell culture maintenance, Dr. Elena Novelli (IN-CNR, Pisa) for assistance in confocal microscopy, Allegra Coppini, Daniele Ferrigno, Jacopo Elia Catastini, Silvia Laria, Eleonora Landi, Matteo Matteucci, and Guglielma De Matienzo (UNIPI) for technical support and quantitative analyses.

CONFLICT OF INTERESTS

The authors declare no competing interests.

Received: May 28, 2021
Revised: May 13, 2022
Accepted: May 17, 2022
Published: June 16, 2022

REFERENCES

Baek, S.T., Copeland, B., Yun, E.-J., Kwon, S.-K., Guemez-Gamboa, A., Schaffer, A.E., Kim, S., Kang, H.-C., Song, S., Mathern, G.W., et al. (2015). An AKT3-FOXG1-reelin network underlies defective migration in human focal malformations of cortical development. Nat. Med. *21*, 1445–1454. https://doi.org/10.1038/nm.3982.

Baggiani, M., Dell'Anno, M.T., Pistello, M., Conti, L., and Onorati, M. (2020). Human neural stem cell systems to explore pathogen-related neurodevelopmental and neurodegenerative disorders. Cells *9*, 1893. https://doi.org/10.3390/cells9081893.

Blackmon, K., Waechter, R., Landon, B., Noël, T., Macpherson, C., Donald, T., Cudjoe, N., Evans, R., Burgen, K.S., Jayatilake, P., et al. (2020). Epilepsy surveillance in normocephalic children with and without prenatal Zika virus exposure. PLoS Neglected Trop. Dis. *14*, e0008874. https://doi.org/10.1371/journal.pntd.0008874.

Boggio, E.M., Pancrazi, L., Gennaro, M., lo Rizzo, C., Mari, F., Meloni, I., Ariani, F., Panighini, A., Novelli, E., Biagioni, M., et al. (2016). Visual impairment in FOXG1-mutated individuals and mice. Neuroscience *324*, 496–508. https://doi.org/10.1016/j.neuroscience.2016.03.027.

Bulstrode, H., Johnstone, E., Marques-Torrejon, M.A., Ferguson, K.M., Bressan, R.B., Blin, C., Grant, V., Gogolok, S., Gangoso, E., Gagrica, S., et al. (2017). Elevated FOXG1 and SOX2 in glioblastoma enforces neural stem cell identity through transcriptional control of cell cycle and epigenetic regulators. Genes Dev. *31*, 757–773. https://doi.org/10.1101/gad.293027.116.

Butler, D. (2016). Zika virus: Brazil's surge in small-headed babies questioned by report. Nature *530*, 13–14. https://doi.org/10.1038/nature.2016.19259.

Cargnin, F., Kwon, J.-S., Katzman, S., Chen, B., Lee, J.W., and Lee, S.-K. (2018). FOXG1 orchestrates neocortical organization and cortico-cortical connections. Neuron *100*, 1083–1096.e5. https://doi.org/10.1016/j.neuron.2018.10.016.

Carlin, C.R. (2022). Role of EGF receptor regulatory networks in the host response to viral infections. Front. Cell. Infect. Microbiol. *11*, 820355. https://doi.org/10.3389/fcimb.2021.820355.

Dali, R., Verginelli, F., Pramatarova, A., Sladek, R., and Stifani, S. (2018). FOXG1:TLE1 transcriptional network in glioblastoma-initiating cells. Mol. Oncol. *12*, 775–787. https://doi.org/10.1002/1878-0261.12168.

Dastidar, S.G., Landrieu, P.M.Z., and D'Mello, S.R. (2011). FoxG1 promotes the survival of postmitotic neurons. J. Neurosci. *31*, 402–413. https://doi.org/10.1523/JNEUROSCI.2897-10.2011.

Dell' Amico, C., Tata, A., Pellegrino, E., Onorati, M., and Conti, L. (2021). Genome editing in stem cells for genetic neurodisorders. In Curing Genetic Diseases Through Genome Reprogramming, G. Petris, ed. (Academic Press), pp. 403–438.

D'Orsi, B., Engel, T., Pfeiffer, S., Nandi, S., Kaufmann, T., Henshall, D.C., and Prehn, J.H.M. (2016). Bok is not pro-apoptotic but suppresses poly ADP-ribose polymerase-dependent cell death pathways and protects against excitotoxic and seizure-induced neuronal injury. J. Neurosci. *36*, 4564–4578. https://doi.org/10.1523/JNEUROSCI.3780-15.2016.

Faria, N.R., Azevedo, R.d.S.d.S., Kraemer, M.U.G., Souza, R., Cunha, M.S., Hill, S.C., Thézé, J., Bonsall, M.B., Bowden, T.A., Rissanen, I., et al. (2016). Zika virus in the Americas: early epidemiological and genetic findings. Science *352*, 345–349. https://doi.org/10.1126/SCIENCE.AAF5036.

de Filippis, R., Pancrazi, L., Bjørgo, K., Rosseto, A., Kleefstra, T., Grillo, E., Panighini, A., Cardarelli, F., Meloni, I., Ariani, F., et al. (2012). Expanding the phenotype associated with FOXG1 mutations and in vivo FoxG1 chromatin-binding dynamics. Clin.

Genet. *82*, 395–403. https://doi.org/10.1111/j.1399-0004.2011.01810.x.

Florian, C., Bahi-Buisson, N., and Bienvenu, T. (2012). FOXG1-Related disorders: from clinical description to molecular genetics. Mol. Syndromol. *2*, 153–163. https://doi.org/10.1159/000327329.

Focosi, D., Maggi, F., and Pistello, M. (2016). Zika virus: implications for public health. Clin. Infect. Dis. *63*, 227–233. https://doi.org/10.1093/cid/ciw210.

Graham, V., Khudyakov, J., Ellis, P., and Pevny, L. (2003). SOX2 functions to maintain neural progenitor identity. Neuron *39*, 749–765. https://doi.org/10.1016/S0896-6273(03)00497-5.

Hammack, C., Ogden, S.C., Madden, J.C., Medina, A., Xu, C., Phillips, E., Son, Y., Cone, A., Giovinazzi, S., Didier, R.A., et al. (2019). Zika virus infection induces DNA damage response in human neural progenitors that enhances viral replication. J. Virol. *93*, 638–657. https://doi.org/10.1128/JVI.00638-19.

Hanashima, C., Shen, L., Li, S.C., and Lai, E. (2002). Brain factor-1 controls the proliferation and differentiation of neocortical progenitor cells through independent mechanisms. J. Neurosci. *22*, 6526–6536. https://doi.org/10.1523/JNEUROSCI.22-15-06526.2002.

Hettige, N.C., and Ernst, C. (2019). FOXG1 dose in brain development. Front. Pediatr. *7*, 482. https://doi.org/10.3389/fped.2019.00482.

Heymann, D.L., Hodgson, A., Sall, A.A., Freedman, D.O., Staples, J.E., Althabe, F., Baruah, K., Mahmud, G., Kandun, N., Vasconcelos, P.F.C., et al. (2016). Zika virus and microcephaly: why is this situation a PHEIC? Lancet *387*, 719–721. https://doi.org/10.1016/S0140-6736(16)00320-2.

Hou, P.-S., HAilín, D.Ó., Vogel, T., and Hanashima, C. (2020). Transcription and beyond: delineating FOXG1 function in cortical development and disorders. Front. Cell. Neurosci. *14*, 35. https://doi.org/10.3389/fncel.2020.00035.

Jiang, X., Dong, X., Li, S.-H., Zhou, Y.-P., Rayner, S., Xia, H.-M., Gao, G.F., Yuan, H., Tang, Y.-P., and Luo, M.-H. (2018). Proteomic analysis of Zika virus infected primary human fetal neural progenitors suggests a role for doublecortin in the pathological consequences of infection in the cortex. Front. Microbiol. *9*, 1067. https://doi.org/10.3389/fmicb.2018.01067.

Kaestner, K.H., Knochel, W., and Martinez, D.E. (2000). Unified nomenclature for the winged helix/forkhead transcription factors. Genes Dev. *14*, 142–146. https://doi.org/10.1101/gad.14.2.142.

Kam, Y.-W., Leite, J.A., Lum, F.-M., Tan, J.J.L., Lee, B., Judice, C.C., Teixeira, D.A.d.T., Andreata-Santos, R., Vinolo, M.A., Angerami, R., et al. (2017). Specific biomarkers associated with neurological complications and congenital central nervous system Abnormalities from Zika virus–infected patients in Brazil. J. Infect. Dis. *216*, 172–181. https://doi.org/10.1093/infdis/jix261.

Kan, L., Jalali, A., Zhao, L.-R., Zhou, X., McGuire, T., Kazanis, I., Episkopou, V., Bassuk, A.G., and Kessler, J.A. (2007). Dual function of Sox1 in telencephalic progenitor cells. Dev. Biol. *310*, 85–98. https://doi.org/10.1016/j.ydbio.2007.07.026.

Kumamoto, T., and Hanashima, C. (2017). Evolutionary conservation and conversion of Foxg1 function in brain development. Dev. Growth Differ. *59*, 258–269. https://doi.org/10.1111/dgd.12367.

Lai, M., Iacono, E., Spezia, P.G., Lottini, G., la Rocca, V., Quaranta, P., Pistello, M., and Freer, G. (2022). A low-cost simple test for weekly detection of Mycoplasma hyorhinis and arginini contaminations in cell cultures and viral preparations. J. Virol Methods *299*, 114327. https://doi.org/10.1016/j.jviromet.2021.114327.

Langford, D., Hurford, R., Hashimoto, M., Digicaylioglu, M., and Masliah, E. (2005). Signalling crosstalk in FGF2-mediated protection of endothelial cells from HIV-gp120. BMC Neurosci. *6*, 8. https://doi.org/10.1186/1471-2202-6-8.

Ledur, P.F., Karmirian, K., Pedrosa, C.d.S.G., da, S.G., Souza, L.R.Q., Assis-de-Lemos, G., Ferreira, J.d.C.C. G., Ferreira, J. de C.C.G., de Azevedo Reis, G.F., Silva, E.S., et al. (2020). Zika virus infection leads to mitochondrial failure, oxidative stress and DNA damage in human iPSC-derived astrocytes. Sci. Rep. *10*, 1218. https://doi.org/10.1038/s41598-020-57914-x.

Li, H., Saucedo-Cuevas, L., Yuan, L., Ross, D., Johansen, A., Sands, D., Stanley, V., Guemez-Gamboa, A., Gregor, A., Evans, T., et al. (2019). Zika virus protease cleavage of host protein septin-2 mediates mitotic defects in neural progenitors. Neuron *101*, 1089–1098.e4. https://doi.org/10.1016/j.neuron.2019.01.010.

Limonta, D., Jovel, J., Kumar, A., Lu, J., Hou, S., Airo, A.M., Lopez-Orozco, J., Wong, C.P., Saito, L., Branton, W., et al. (2019). Fibroblast growth factor 2 enhances Zika virus infection in human fetal brain. J. Infect. Dis. *220*, 1377–1387. https://doi.org/10.1093/infdis/jiz073.

Maddaluno, L., Urwyler, C., Rauschendorfer, T., Meyer, M., Stefanova, D., Spörri, R., Wietecha, M., Ferrarese, L., Stoycheva, D., Bender, D., et al. (2020). Antagonism of interferon signaling by fibroblast growth factors promotes viral replication. EMBO Mol. Med. *12*, e11793. https://doi.org/10.15252/emmm.201911793.

Marcal, N., Patel, H., Dong, Z., Belanger-Jasmin, S., Hoffman, B., Helgason, C.D., Dang, J., and Stifani, S. (2005). Antagonistic effects of Grg6 and groucho/TLE on the transcription repression activity of brain factor 1/FoxG1 and cortical neuron differentiation. Mol. Cell Biol. *25*, 10916–10929. https://doi.org/10.1128/MCB.25.24.10916-10929.2005.

Mariani, J., Coppola, G., Zhang, P., Abyzov, A., Provini, L., Tomasini, L., Amenduni, M., Szekely, A., Palejev, D., Wilson, M., et al. (2015). FOXG1-Dependent dysregulation of GABA/glutamate neuron differentiation in autism spectrum disorders. Cell *162*, 375–390. https://doi.org/10.1016/j.cell.2015.06.034.

Mitter, D., Pringsheim, M., Kaulisch, M., Plümacher, K.S., Schröder, S., Warthemann, R., Abou Jamra, R., Baethmann, M., Bast, T., Büttel, H.-M., et al. (2018). FOXG1 syndrome: genotype–phenotype association in 83 patients with FOXG1 variants. Genet. Med. *20*, 98–108. https://doi.org/10.1038/gim.2017.75.

Morazzani, E.M., Compton, J.R., Leary, D.H., Berry, A.v., Hu, X., Marugan, J.J., Glass, P.J., and Legler, P.M. (2019). Proteolytic cleavage of host proteins by the Group IV viral proteases of Venezuelan equine encephalitis virus and Zika virus. Antivir. Res. *164*, 106–122. https://doi.org/10.1016/j.antiviral.2019.02.001.

Musso, D., and Gubler, D.J. (2016). Zika virus. Clin. Microbiol. Rev. *29*, 487–524. https://doi.org/10.1128/CMR.00072-15.

Obendorf, M., Meyer, R., Henning, K., Mitev, Y.A., Schröder, J., Patchev, V.K., and Wolf, S.S. (2007). FoxG1, a member of the

forkhead family, is a corepressor of the androgen receptor. J. Steroid Biochem. Mol. Biol. *104*, 195–207. https://doi.org/10.1016/j.jsbmb.2007.03.012.

Onorati, M., Castiglioni, V., Biasci, D., Cesana, E., Menon, R., Vuono, R., Talpo, F., Laguna Goya, R., Lyons, P.A., Bulfamante, G.P., et al. (2014). Molecular and functional definition of the developing human striatum. Nat. Neurosci. *17*, 1804–1815. https://doi.org/10.1038/nn.3860.

Onorati, M., Li, Z., Liu, F., Sousa, A.M.M., Nakagawa, N., Li, M., Dell'Anno, M.T., Gulden, F.O., Pochareddy, S., Tebbenkamp, A.T.N., et al. (2016). Zika virus disrupts phospho-TBK1 localization and mitosis in human neuroepithelial stem cells and radial glia. Cell Rep. *16*, 2576–2592. https://doi.org/10.1016/j.celrep.2016.08.038.

Pancrazi, L., di Benedetto, G., Colombaioni, L., della Sala, G., Testa, G., Olimpico, F., Reyes, A., Zeviani, M., Pozzan, T., and Costa, M. (2015). Foxg1 localizes to mitochondria and coordinates cell differentiation and bioenergetics. Proc. Natl. Acad. Sci. U S A *112*, 13910–13915. https://doi.org/10.1073/pnas.1515190112.

Qian, X., Nguyen, H.N., Song, M.M., Hadiono, C., Ogden, S.C., Hammack, C., Yao, B., Hamersky, G.R., Jacob, F., Zhong, C., et al. (2016). Brain-region-specific organoids using mini-bioreactors for modeling ZIKV exposure. Cell *165*, 1238–1254. https://doi.org/10.1016/j.cell.2016.04.032.

Regad, T., Roth, M., Bredenkamp, N., Illing, N., and Papalopulu, N. (2007). The neural progenitor-specifying activity of FoxG1 is antagonistically regulated by CKI and FGF. Nat. Cell Biol. *9*, 531–540. https://doi.org/10.1038/ncb1573.

Rothan, H.A., Fang, S., Mahesh, M., and Byrareddy, S.N. (2019). Zika virus and the metabolism of neuronal cells. Mol. Neurobiol. *56*, 2551–2557. https://doi.org/10.1007/s12035-018-1263-x.

Seoane, J., Le, H.-V., Shen, L., Anderson, S.A., and Massagué, J. (2004). Integration of smad and forkhead pathways in the control of neuroepithelial and glioblastoma cell proliferation. Cell *117*, 211–223. https://doi.org/10.1016/S0092-8674(04)00298-3.

Sousa, A.M.M., Zhu, Y., Raghanti, M.A., Kitchen, R.R., Onorati, M., Tebbenkamp, A.T.N., Stutz, B., Meyer, K.A., Li, M., Kawasawa, Y.I., et al. (2017). Molecular and cellular reorganization of neural circuits in the human lineage. Science *358*, 1027–1032. https://doi.org/10.1126/science.aan3456.

Tan, K., Shaw, A.L., Madsen, B., Jensen, K., Taylor-Papadimitriou, J., and Freemont, P.S. (2003). Human PLU-1 has transcriptional repression properties and interacts with the developmental transcription factors BF-1 and PAX9. J. Biol. Chem. *278*, 20507–20513. https://doi.org/10.1074/jbc.M301994200.

Tang, H., Hammack, C., Ogden, S.C., Wen, Z., Qian, X., Li, Y., Yao, B., Shin, J., Zhang, F., Lee, E.M., et al. (2016). Zika virus infects human cortical neural progenitors and attenuates their growth. Cell Stem Cell *18*, 587–590. https://doi.org/10.1016/j.stem.2016.02.016.

Vasudevan, J., Skandhan, A., Skandhan, A.K.P., Balakrishnan, S., Skandhan, K.P., and Skandhand, K.P. (2018). Zika virus. Rev. Med. Microbiol. *29*, 43–50. https://doi.org/10.1097/MRM.0000000000000126.

VHP, L., Aragão, M., Pinho, R., Hazin, A., Paciorkowski, A., Penalva de Oliveira, A., and Masruha, M.R. (2020). Congenital Zika virus infection: a review with emphasis on the spectrum of brain abnormalities. Curr. Neurol. Neurosci. Rep. *20*, 49. https://doi.org/10.1007/s11910-020-01072-0.

Wong, L.-C., Singh, S., Wang, H.-P., Hsu, C.-J., Hu, S.-C., and Lee, W.-T. (2019). FOXG1-Related syndrome: from clinical to molecular genetics and pathogenic mechanisms. Int. J. Mol. Sci. *20*, 4176. https://doi.org/10.3390/ijms20174176.

Xiong, Y., Zhang, Y., Xiong, S., and Williams-Villalobo, A.E. (2020). A glance of p53 functions in brain development, neural stem cells, and brain cancer. Biology *9*, 285. https://doi.org/10.3390/biology9090285.

Yang, S., Gorshkov, K., Lee, E.M., Xu, M., Cheng, Y.-S., Sun, N., Soheilian, F., de Val, N., Ming, G., Song, H., et al. (2020). Zika virus-induced neuronal apoptosis via increased mitochondrial fragmentation. Front. Microbiol. *11*, 3316. https://doi.org/10.3389/fmicb.2020.598203.

Zhang, Z., Rong, L., and Li, Y.-P. (2019). *Flaviviridae* viruses and oxidative stress: implications for viral pathogenesis. Oxid. Med. Cell. Longev. *2019*, 1–17. https://doi.org/10.1155/2019/1409582.

Zhao, X., Bian, R., Wang, F., Wang, Y., Li, X., Guo, Y., Zhang, X., Luo, G., and Zhan, R. (2021). GDF-5 promotes epidermal stem cells proliferation via Foxg1-cyclin D1 signaling. Stem Cell Res. Ther. *12*, 42. https://doi.org/10.1186/s13287-020-02106-7.

Zhu, Z., Gorman, M.J., McKenzie, L.D., Chai, J.N., Hubert, C.G., Prager, B.C., Fernandez, E., Richner, J.M., Zhang, R., Shan, C., et al. (2017). Zika virus has oncolytic activity against glioblastoma stem cells. J. Exp. Med. *214*, 2843–2857. https://doi.org/10.1084/jem.20171093.

Stem Cell Reports, Volume *17*

Supplemental Information

Zika virus induces FOXG1 nuclear displacement and downregulation in human neural progenitors

Giulia Lottini, Matteo Baggiani, Giulia Chesi, Beatrice D'Orsi, Paola Quaranta, Michele Lai, Laura Pancrazi, Marco Onorati, Mauro Pistello, Giulia Freer, and Mario Costa

Supplemental items

Figure S1

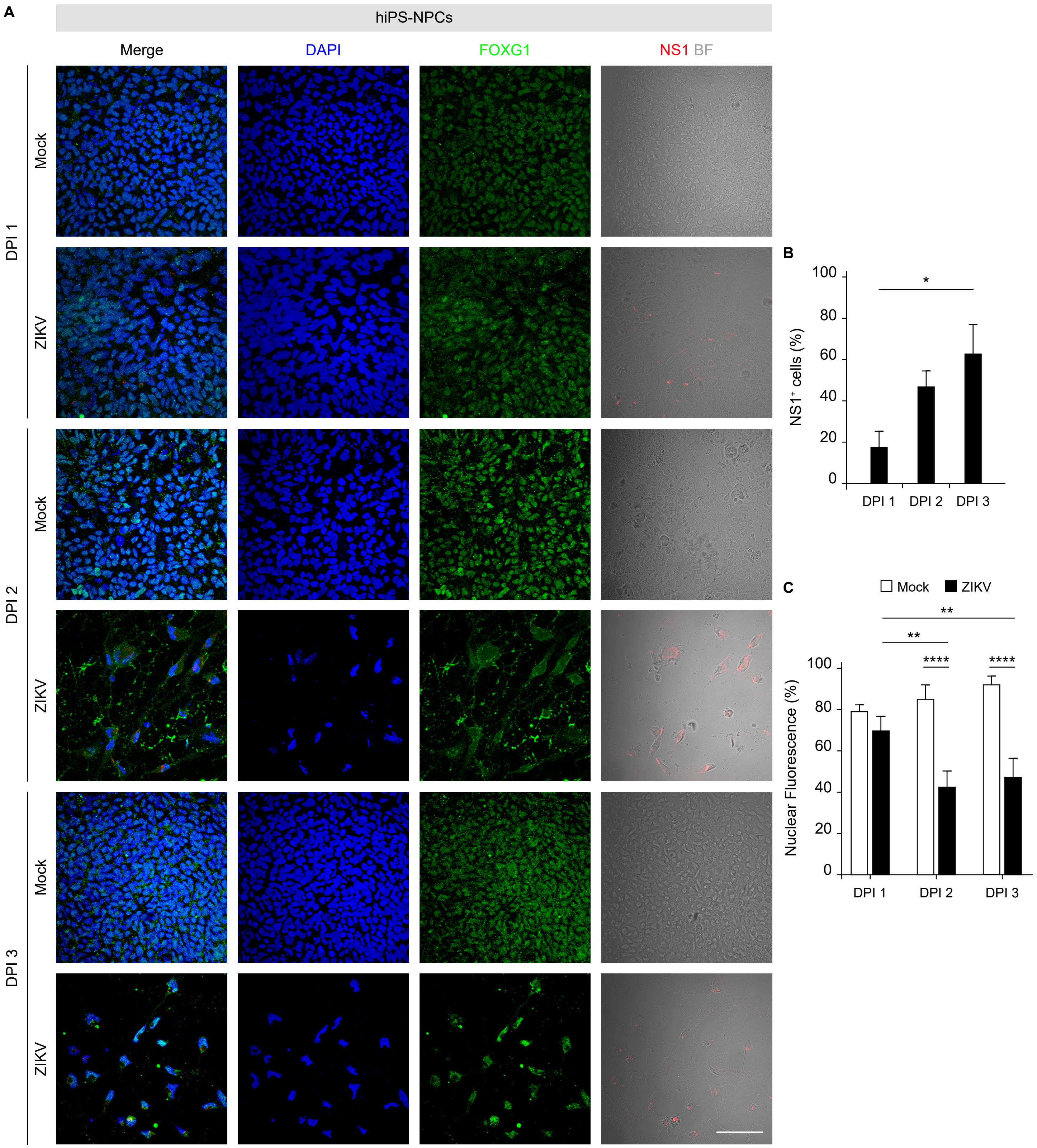

Figure S1. Infectivity rate and FOXG1 mislocalization in hiPS-NPCs after ZIKV infection, Related to Figure 1.
(A) Representative confocal images of FOXG1, ZIKV NS1, Bright Field (BF), and DAPI in mock and ZIKV-infected hiPS-NPCs at day post-infection (DPI) 1, 2, and 3. The time-course analysis shows progressive ZIKV infection and FOXG1 mislocalization from DPI 1 to 3. Scale bar = 50 µm. (B) Bar plot indicating the rate of ZIKV infection at each DPI. Data are shown as mean ± SEM (total cells, n > 800), *p*-value < 0.05 (One-way ANOVA, *post hoc* Tukey's test). (C) Bar plot indicating the ratio of FOXG1 nuclear fluorescence on total fluorescence in mock and ZIKV-infected conditions at each DPI. Data are shown as mean ± SD (total cells, n = 120), *p*-value < 0.01 (Two-way ANOVA, *post hoc* Tukey's test).

Figure S2

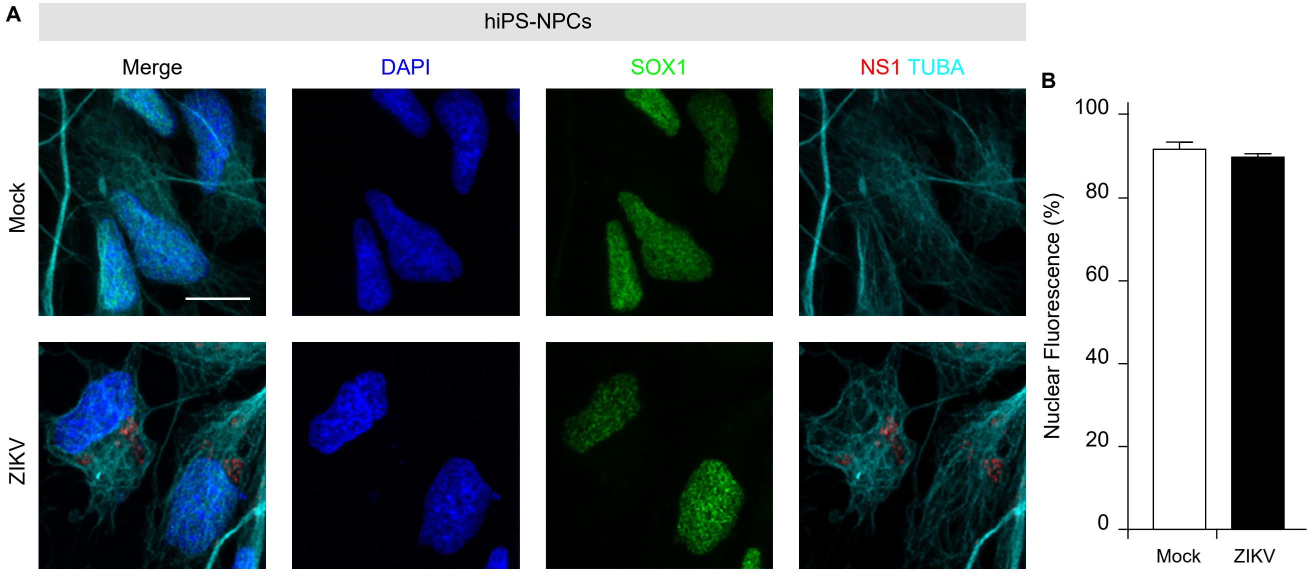

Figure S2. ZIKV infection does not induce mislocalization of SOX1 in hiPS-NPCs, Related to Figure 1. (A) Representative confocal images of SOX1, ZIKV NS1, TUBA (α-tubulin), and DAPI in mock and ZIKV-infected hiPS-NPCs. Scale bar = 10 µm. (B) Bar plot indicating the ratio of SOX1 nuclear fluorescence on total fluorescence in mock and ZIKV-infected conditions. Data are shown as mean ± SD (total cells, n = 40), *p*-value > 0.05 (unpaired Student's *t*-test).

Figure S3

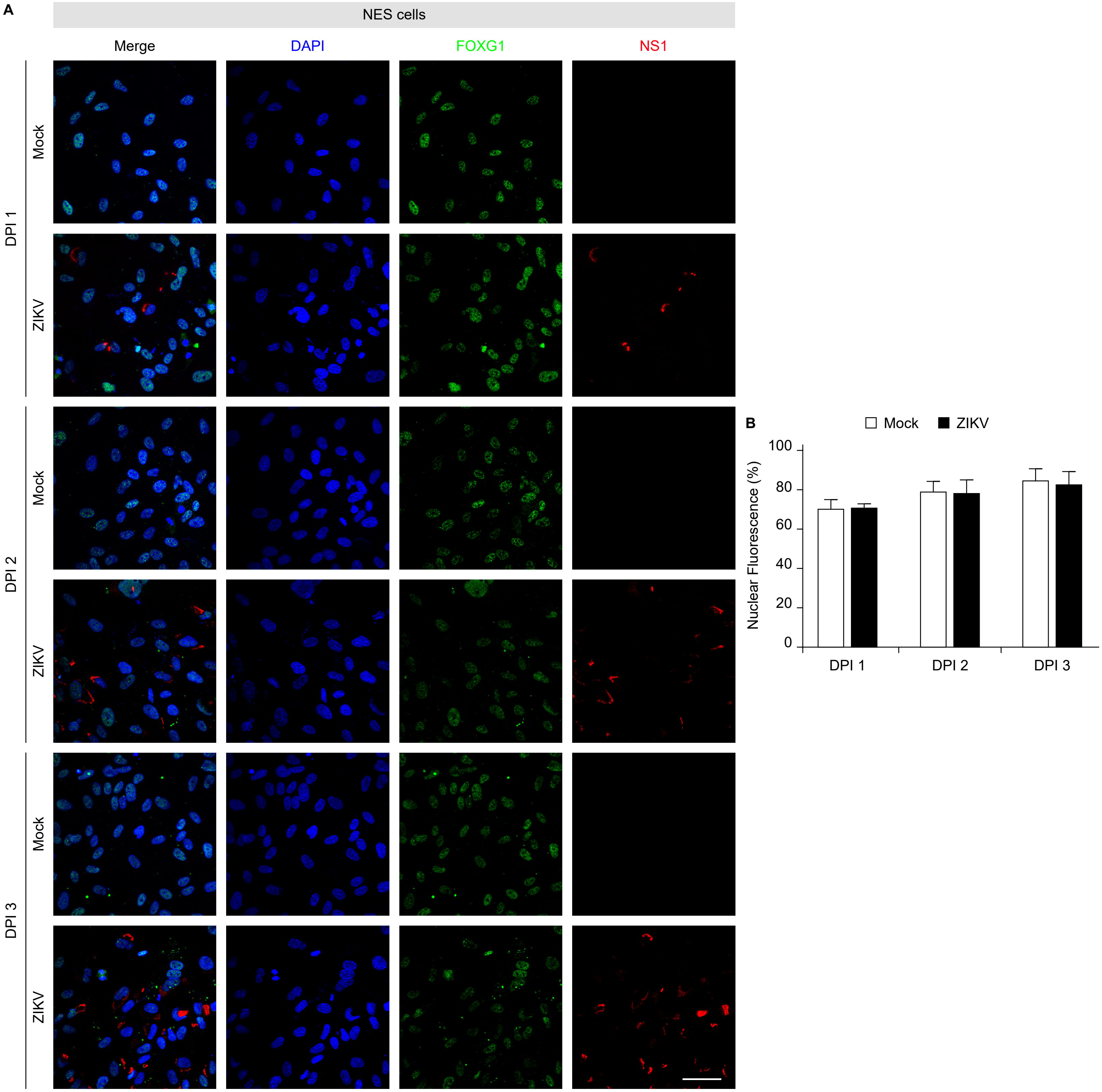

Figure S3. Time-course of FOXG1 localization pattern in NES cells after ZIKV infection, Related to Figure 3.
(A) Representative confocal images of FOXG1, ZIKV NS1, and DAPI in mock and ZIKV-infected NES cells at DPI 1, 2, and 3. The time course analysis shows that FOXG1 does not mislocalize from the nucleus. Scale bar = 50 μm. (B) Bar plot indicates the ratio of FOXG1 nuclear fluorescence on total fluorescence in mock and ZIKV-infected conditions at each DPI. Data are shown as mean ± SD (total cells, n = 120), *p*-value > 0.05 (Two-way ANOVA, *post hoc* Tukey's test).

Figure S4

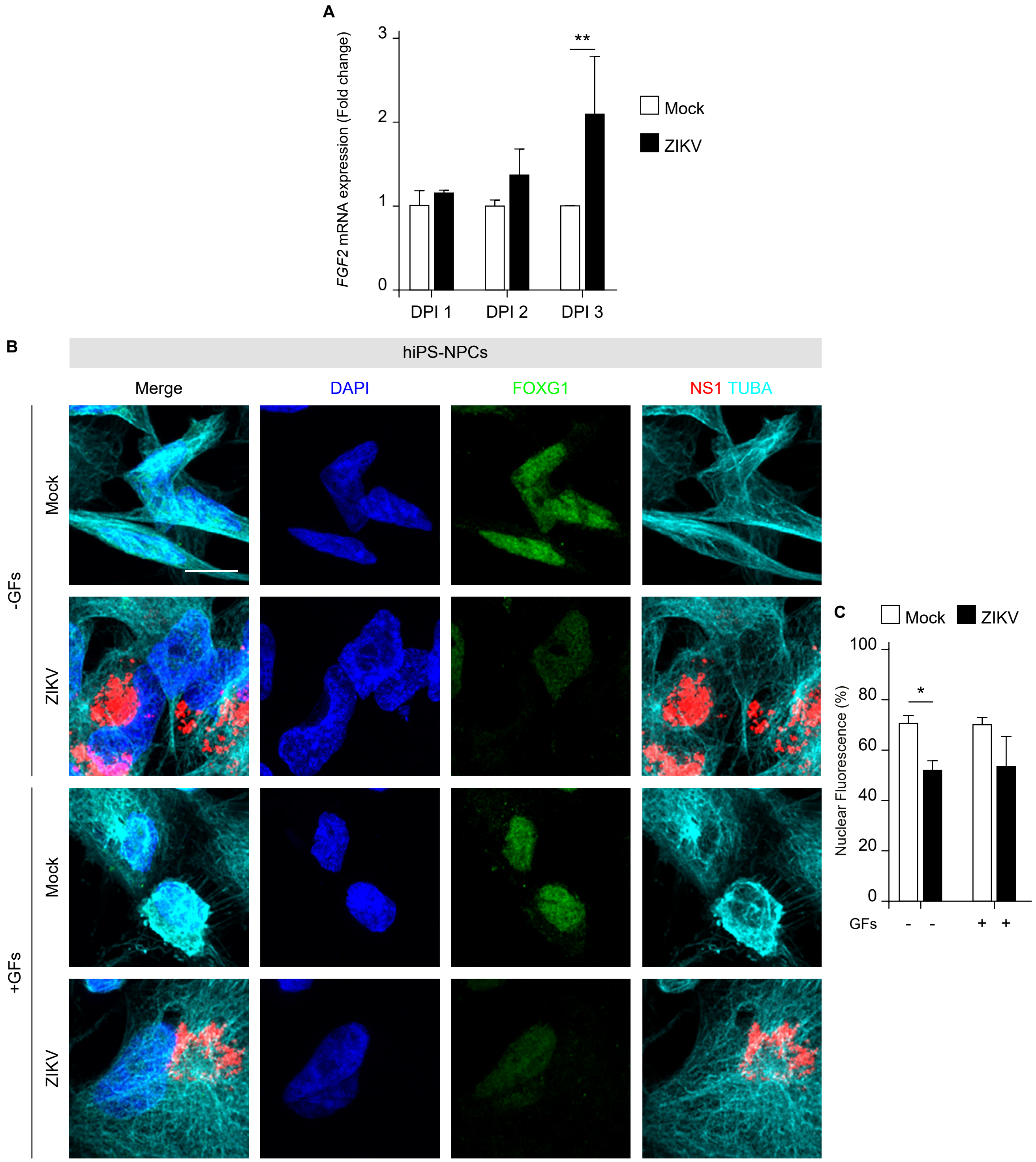

Figure S4. *FGF2* expression analysis and GF treatment after ZIKV infection in hiPS-NPCs, Related to Figure 3.

(A) Bar plot indicating fold change in *FGF2* mRNA level in mock and ZIKV-infected conditions. Data are shown as mean ± SD (n = 3), *p*-value < 0.01 (Two-way ANOVA, *post hoc* Tukey's test). (B) Representative confocal images of FOXG1, ZIKV NS1, TUBA (α-tubulin), and DAPI in mock and ZIKV-infected hiPS-NPCs in the absence and in the presence of GFs (EGF and FGF2) after the infection, in which FOXG1 preserves nuclear localization following ZIKV infection only in presence of GFs. Analyses were performed at DPI 3. Scale bar = 10 μm. (C) Bar plot indicating the ratio of FOXG1 nuclear fluorescence on total fluorescence in mock and ZIKV-infected conditions at DPI 3. Data are shown as mean ± SD (total cells, n = 80), *p*-value < 0.05 (Two-way ANOVA, *post hoc* Tukey's test).

Figure S5

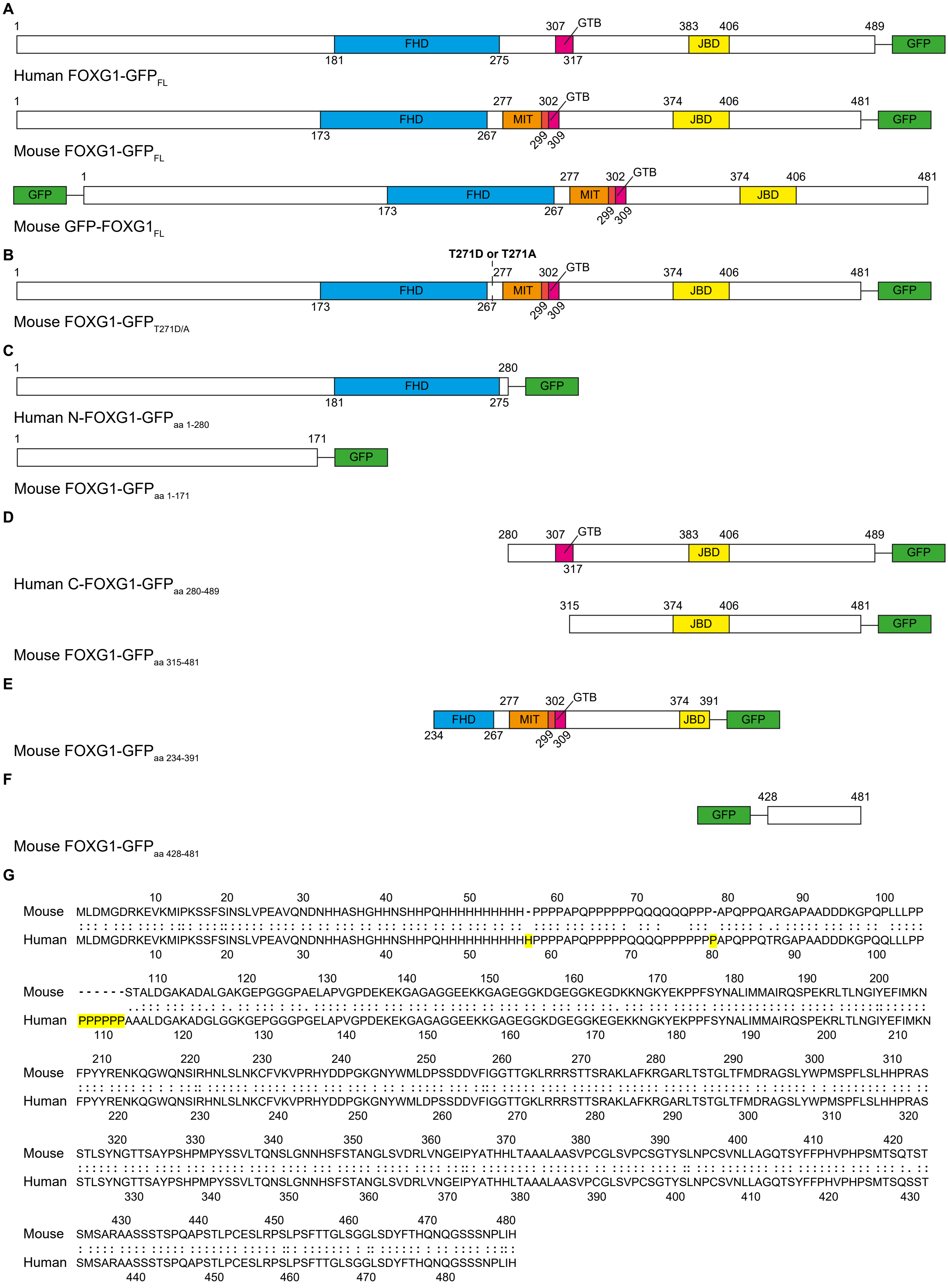

Figure S5. Human and mouse FOXG1-GFP constructs, Related to Figures 4 and 5.
Schematic illustration of (A) human FOXG1-GFP full length (FL), mouse FOXG1-GFP FL and mouse GFP-FOXG1 FL; (B) mutated mouse FOXG1-GFP on Thr271 (T271D/A); (C) N-terminal human FOXG1-GFP (1-280) and N-terminal mouse FOXG1-GFP (1-171); (D) C-terminal human FOXG1-GFP (280-489) and C-terminal mouse FOXG1-GFP (315-481); (E) central domains of mouse FOXG1-GFP (234-391) (F) final part of C-terminal mouse FOXG1-GFP (428-481). (G) Comparison between mouse and human aa sequences of FOXG1; the human-specific aa in FOXG1 protein are highlighted in yellow. FHD = Forkhead Domain (blue); MIT = Mitochondrial domain (orange); GTB = GROUCHO/TLE-Binding domain (pink); JBD = JARID1B Binding Domain (yellow); GFP = Green Fluorescence Protein (green).

Figure S6

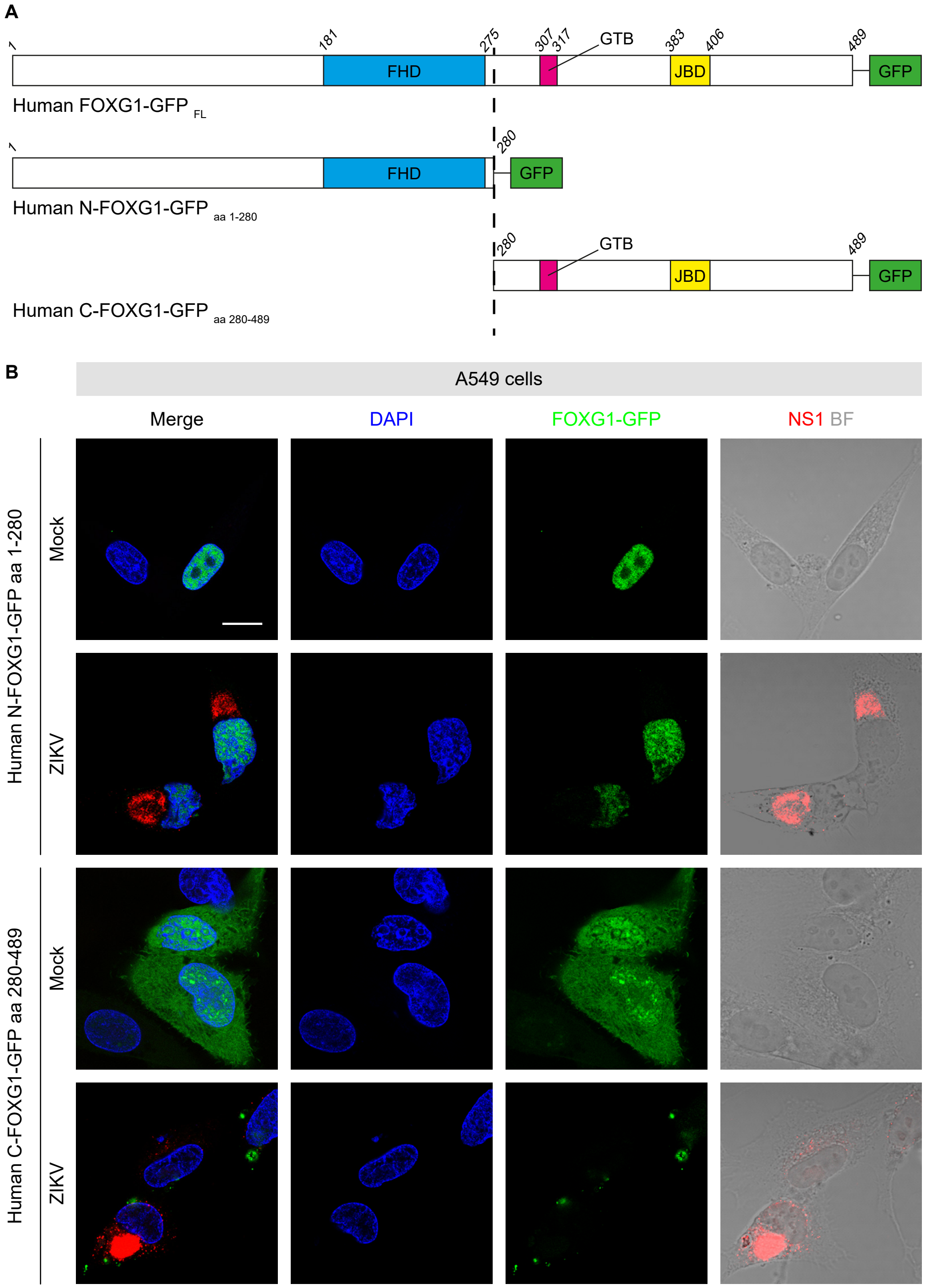

Figure S6. Human FOXG1 C-terminus is essential for reacting to ZIKV infection, Related to Figure 5.

(A) Schematic illustration of FOXG1-GFP full length (FL) and the partial constructs (human N-FOXG1-GFP aa 1-280; human C-FOXG1-GFP aa 280-489). FHD = Forkhead Domain (blue); GTB = GROUCHO/TLE-Binding domain (pink); JBD = JARID1B Binding Domain (yellow); GFP = Green Fluorescence Protein (green). (B) Representative confocal images of human N-FOXG1-GFP aa 1-280 and human C-FOXG1-GFP aa 280-489 transfected A549 cells in mock and ZIKV-infected conditions. BF = Bright field. Analyses were performed at DPI 1. Scale bar = 20 μm.

Supplemental experimental procedures

hiPS-NPC maintenance and derivation

We derived neural progenitor cells from hiPSCs, as previously reported (1). Briefly, hiPSCs were dissociated into single cells in StemFlex medium (Thermo Fisher Scientific; #A3349201)] in Matrigel coated dishes containing 10 µM Y-27632, until confluent. Then, we performed the dual SMAD inhibition protocol changing the StemFlex medium with a neural induction medium [1:1 Dulbecco's minimum essential medium/F12 (DMEM/F12) (Gibco #11330-032) and Neurobasal medium (Gibco #21103-049) with addition of B27 supplement (1:50, Gibco, #175040-44), N2 supplement (1:100, Gibco, #17502-048), 20 µg/ml insulin (Sigma, # I9278), L-glutamine (1:100, Gibco, #25030-081), MEM Non-Essential Amino Acids (1:100, Gibco, #11140-050) and 2-mercaptoethanol (1:1000, Gibco, #21985)], supplemented with 100 nM of LDN-193189 (StemCell Technologies, # 72144), 10 µM of SB-431542 (Merck, # 616464-5MG) and 2 µM of XAV939 (StemCell Technologies, # 72674). The medium was changed daily until day 11. At day 12, the cells were dissociated with Accutase and maintained in a neural differentiation medium [Neurobasal medium (Gibco #21103-049) with addition of B27 supplement (1:50, Gibco, #175040-44), N2 supplement (1:100, Gibco, #17502-048), L-glutamine (1:100, Gibco, #25030-081), with Y-27632 (10 µM), to increase cell viability.

Maintenance of NES cells

Human neuroepithelial stem (NES) cells were cultured as already reported (2,3). Briefly, NES cells were maintained in NES medium [Dulbecco's minimum essential medium/F12 (DMEM/F12) (Gibco #11330-032) with addition of B27 supplement (1:1000, Gibco, #175040-44), N2 supplement (1:100, Gibco, #17502-048), 20 ng/ml FGF-2 (Gibco, #13256029), 20 ng/ml EGF (Gibco, #PHG0311), 1.6 g/l glucose, 20 µg/ml insulin (Sigma, # I9278) and 5 ng/ml BDNF (Gibco, #PHC7074)] in poly-L-ornithine (0.01%, Sigma, #P4957), laminin (5 µg/ml, Invitrogen #23017-015) and fibronectin (1µg/ml, Corning, #354008) coated dishes. In order to preserve their optimal growth and neurogenic properties, the medium should be changed every 2-3 days and the cells should be passaged 1:2-3 when they are confluent, once every 5-7 days (~0.5–1 x 105 cells/cm2).

Maintenance of A549 cells

A549 were grown in DMEM, high glucose, 1 mM glutamine, 10% FCS, unless otherwise stated. No antibiotics were added. All the cells were routinely tested for mycoplasma (4).

DNA constructs

Mouse *Foxg1* aa 1-171cDNA was amplified by PCR using *GFP-Foxg1* wt as DNA template and a forward primer incorporating the *XhoI* restriction site that occurs in the *Foxg1* cDNA (underlined) Forward primer: 5'-ACTCGAGCATGCTGGACATGGGAGATAGG-3'.
The reverse primer was 5'GGATCCCCATGTATTAAAGGGTTGGAAG-3', incorporating a *BamHI* restriction site (underlined). The amplification product was purified and cleaved with *XhoI/BamHI* and ligated to the corresponding restriction sites in the vector pEGFP-N1 (Clontech, USA). FOXG1 aa 1-280-GFP, FOXG1 aa 280-489-GFP, FOXG1- T271D-GFP (phospho-mimetic T271-ACG were changed in Asp-GAC), FOXG1-T271A-GFP (phospho- defective T271-ACG were changed in Ala-GCG) were purchased at IDT, Belgium. Mouse *Foxg1* aa 315-489 cDNA was amplified by PCR using *GFP-Foxg1* as DNA template. The forward primer was 5'-GGTACCAATGAGCACTTTGAGTTACAACGG-3', incorporating a *KpnI* restriction site (underlined) and a start codon (bold) before the codon coding for amino acid 315. The reverse primer was 5'-GGATCCCCATGTATTAAAGGGTTGGAAG-3', incorporating a *BamHI* restriction site (underlined). The amplification product was purified and cleaved with *KpnI/BamHI* and ligated to the corresponding restriction sites in the vector pEGFP-N1 (Clontech, USA). The GFP-FOXG1 aa 428-481 construct was generated digesting *Foxg1-GFP* with *SmaI* and *BamHI.* The excised fragment was cloned in frame into the corresponding restriction sites of pEGFP-C2.

Immunofluorescence

Cultured cells were fixed in 4% formaldehyde (FA) for 12 min at RT. After two 3-minutes washed with Phosphate Buffered Saline-Triton X-100 (PBSX) [0.1% (vol/vol) Triton X-100 in PBS Ca2+/Mg2+ 1X] cells were permeabilized for 10 min with permeabilization solution [0.5% (vol/vol) Triton X-100 in PBS Ca2+/Mg2+1X] and blocked for 1 h with blocking solution [5% fetal bovine serum, 0.3% (vol/vol) Triton X-100 in PBS Ca2+/Mg2+1X]. Cells were incubated with primary antibodies against flavivirus NS1 protein (ab214337, [D/2/D6/B7] clone, Abcam, 1:400), FOXG1 (ab214337, Abcam, 1:500), Chikungunya virus native protein (MA5-18181, A54Q clone, Invitrogen, 1:50), Usutu virus native protein (MA5-18281, F50F clone, Invitrogen, 1:50), SOX2 (ab5603, Millipore, 1:400), TUBA (mca77g, Bio-Rad, 1:500), SOX1 (4194, Cell signaling, 1:200), pHH3 (06-570, Millipore, 1:500), cleaved CASP3 (ab3623, Millipore, 1:200) diluted in antibody solution [3% fetal bovine serum, 0.2% (vol/vol) Triton X-100 in PBS Ca2+/Mg2+1X] at 4°C overnight. The next day, cells were washed with PBSX three times for 3 minutes and then incubated for 1h at RT with Alexa Fluor®

secondary antibodies and DAPI (D1306, Invitrogen, 1 μg/ml) diluted in antibody solution. After two washes of 3 minutes with PBSX and one wash of 3 minutes with PBS Ca2+/Mg2+, the fixed cells were mounted on microscope slides to perform confocal analysis. All images were acquired using a laser scanning confocal microscope (Nikon, Eclipse Ti) or a laser scanning confocal microscope Zeiss LSM 9.10 (Carl Zeiss).

Measurement of the nuclear-cytoplasmatic percentage of fluorescence

Using ImageJ software (https://imagej.nih.gov/ij/), a ROI (Region Of Interest) was drawn to select the nucleus and the total cell area (cytoplasm and nucleus) of each cell and to measure the intensity of fluorescence in each selection in the channel of interest. Moreover, one small circle was selected out of each cell to measure the background fluorescence. The following parameters were measured:

- ROI Area: the number of pixels into the ROI
- Mean Gray Value: $\frac{\Sigma\, gray\ intensity\ of\ pixels\ into\ the\ ROI}{number\ of\ pixels\ into\ the\ ROI}$
- Integrated Density: $ROI\ Area \times Mean\ Gray\ Value$

Then, the Corrected Total Cellular Fluorescence (CTCF) was calculated:

- $CTCF_{total} = IntDen_{total} - (ROI\ Area_{\ total}\ x\ Mean\ Gray\ Value_{backgroud})$
- $CTCF_{nuclear} = IntDen_{nuclear} - (ROI\ Area_{\ nuclear}\ x\ Mean\ Gray\ Value_{backgroud})$
- $Nuclear\ fluorescence\ \% = \frac{CTCF_{nuclear}}{CTCF_{total}}\ x\ 100$

Once obtained these values, the target marker nuclear fluorescence of mock and infected cells was compared.

Reverse-transcriptase quantitative PCR (RT-qPCR)

hiPS-NPCs were cultured in a 6-wells cell culture multi-well plate and infected with $ZIKV^{Br.}$ strain with MOI=1 until DPI 3. Total RNA was extracted using the RNeasy Plus mini kit (74134, Qiagen) as per manufacturer instructions. cDNA synthesis was performed using 1 μg of total RNA as the template and reverse transcribed using GoScript™ Reverse Transcriptase (A5001, Promega) primed with 0.50 μg of random hexamers. RT-qPCR was performed using the QuantStudio™ 3 Real-Time PCR System (Applied Biosystems™, A28137) and the SensiMixTM SYBR®No-ROX kit (Meridian BIOSCENCE, QT650-05) as per the manufacturer's protocol. The forward and reverse primers were (5’ to 3’): TGGACGCAGACCTTGAGAAC and GGGCACCTTTACTACGAATGC for *FOXG1*, GCGGAGGAGAACAAAC AGATC and GAGGGCGGATTGGAAATGAAC for *CCND1*, CTCTCAGGGTCGAAAACGGC and GCGGATTAGGGCTTCCTCTTG for *CDKN1A*, CGCAGGAATAAGGAAGCGACC and GGCATTTGGGGAACCGTCTG for *CDKN1B*, and AGCTGAACGGGAAGCTCACT and AGGTCCACCACTGACACGTTG for *GAPDH*. RT-qPCR was performed in 20 µl with the following parameters: an activation step of 95°C for 10 min, followed by 40 cycles of 95°C for 10 sec, 60°C for 15 sec, and 72°C for 15 sec, and a melt curve step of 95°C for 15 sec, 60°C for 1 min, and 95°C for 15 sec. The generation of specific PCR products was confirmed by melting curve analysis. The data were analyzed using the $2^{-\Delta\Delta Ct}$ method with all samples normalized to *GAPDH* and mock condition.

Western blotting

hiPS-NPCs were cultured in a 6-well multi-well plate and infected with $ZIKV^{Br.}$ strain with MOI=1 until DPI 3. Then, cells were lysed in ice with RIPA buffer (R0278, Sigma) with 1% SDS, protease inhibitor 7x (11836170001, Sigma), and phosphatase inhibitor 10x (04906, Roche). They were quantified with DC protein assay (500-0116, Bio-Rad), and boiled at 70°C for 10 min with Laemmli 4x (161-0747, Bio-Rad). Next, lysates were run in Mini-PROTEAN precast gel (4568023, Bio-Rad) in Running buffer at 300V for 15 min and transferred on PVDF membrane (03010040001, Sigma) with Trans-Blot Turbo System (Bio-Rad) at 2.5A for 7 min. The membrane was washed with TBST (0.05% Tween v/v in TBS) for 5 min in rocking plate at RT, incubated with Blocking solution (Milk 5% w/v in TBST) for 1 h in rocking plate at RT, washed with TBST for 2 min in rocking plate at RT, and finally incubated with primary antibodies FOXG1 (ab214337, Abcam, 1:2000), TUBA (3873, Cell signaling, 1:8000), and NS3 (133309, GeneTex, 1:2000) diluted in antibody solution (Milk 1% w/v in TBST) at 4°C overnight in rocking plate. The next day, the membrane was washed 3 times with TBST for 10 min in rocking plate at RT and incubated with secondary antibodies conjugated to peroxidase (SAB3700934, Sigma, 1:2000; SAB3701105, Sigma, 1:2000) diluted in antibody solution for 1h at RT in rocking plate. Finally, after 3 x 10 min-washes in TBST, the membrane was revealed using ECL substrates (170-5060, Bio-Rad) with Chemidoc system. Expected size of western blotting bands: 70 kDa for FOXG1, 69 kDa for NS3, and 50 kDa for TUBA.

Determination of cell death

A549 cells on 24-well plates were stained live with 1 µg/ml Hoechst (33258, Sigma) and 5 µM propidium iodide (PI) (P4170, Sigma) dissolved in culture medium as previously described (5). Nuclear morphology was assessed using an EVOS FLoid Imaging System (Thermofisher, 4471136) inverted microscope with 20X, 0.43 NA phase-contrast objective using the appropriate filter set for Hoechst, PI, GFP and a charge-coupled device camera. The number of PI-positive cells was expressed as a percentage of total cells in the field. Resultant images were processed using Image J software analysis (Wayne Rasband, National Institutes of Health, Bethesda, MD).

References

1. Sousa AMM, Zhu Y, Raghanti MA, Kitchen RR, Onorati M, Tebbenkamp ATN, et al. Molecular and cellular reorganization of neural circuits in the human lineage. Science [Internet]. 2017 Nov 24;358(6366):1027–32. Available from: https://www.sciencemag.org/lookup/doi/10.1126/science.aan3456
2. Dell'Anno MT, Wang X, Onorati M, Li M, Talpo F, Sekine Y, et al. Human neuroepithelial stem cell regional specificity enables spinal cord repair through a relay circuit. Nature Communications. 2018;9(1):3419.
3. Onorati M, Li Z, Liu F, Sousa AMM, Nakagawa N, Li M, et al. Zika Virus Disrupts Phospho-TBK1 Localization and Mitosis in Human Neuroepithelial Stem Cells and Radial Glia. Cell Reports. 2016;16(10):2576–92.
4. Lai M, Iacono E, Spezia PG, Lottini G, La Rocca V, Quaranta P, et al. A low-cost simple test for weekly detection of Mycoplasma hyorhinis and arginini contaminations in cell cultures and viral preparations. Journal of Virological Methods. 2022 Jan 1;299:114327.
5. D'Orsi B, Engel T, Pfeiffer S, Nandi S, Kaufmann T, Henshall DC, et al. Bok Is Not Pro-Apoptotic But Suppresses Poly ADP-Ribose Polymerase-Dependent Cell Death Pathways and Protects against Excitotoxic and Seizure-Induced Neuronal Injury. Journal of Neuroscience [Internet]. 2016 Apr 20 [cited 2021 Dec 9];36(16):4564–78. Available from: https://www.jneurosci.org/lookup/doi/10.1523/JNEUROSCI.3780-15.2016
